# Extracellular Vesicles Derived from Ligament Tissue Transport Interleukin‐17A to Mediate Ligament‐To‐Bone Crosstalk in Ankylosing Spondylitis

**DOI:** 10.1002/advs.202406876

**Published:** 2024-09-23

**Authors:** Kaiyang Wang, Jingshun Lu, Chenyu Song, Mu Qiao, Yao Li, Menghan Chang, Hongda Bao, Yong Qiu, Bang‐Ping Qian

**Affiliations:** ^1^ Division of Spine Surgery Department of Orthopedic Surgery Nanjing Drum Tower Hospital Affiliated Hospital of Medical School Nanjing University Zhongshan Road 321 Nanjing 210008 China

**Keywords:** ankylosing spondylitis, extracellular vesicles, interleukin‐17A, pathological new bone formation

## Abstract

Pathological new bone formation is a critical feature of the progression of ankylosing spondylitis (AS), and spine ankylosis is a distinctive feature of this condition. Ligaments are the primary regions of pathological new bone formation in AS. Here, it is demonstrated that ligament tissue‐derived extracellular vesicles (EVs) and their interleukin‐17A (IL‐17A) cargo mediate the communication between the tissue and other cells. The investigation revealed that IL‐17A in EVs can activate the JAK‐STAT3 pathway, thereby stimulating the expression of MMP14 in AS ligament. Overexpression of MMP14 can lead to changes in the cytoskeleton and mechanical signaling of mesenchymal stem cells and other cells. These alterations in cellular cytoskeleton and mechanical signaling at ligament sites in patients with AS or in stem cells treated with EVs can result in pathological new bone formation. Finally, inhibiting IL‐17A activity and EV endocytosis effectively controlled inflammation and pathological new bone formation. Overall, these data suggest that ligament‐derived EVs and the enclosed IL‐17A have a potential role in driving pathological new bone formation in AS, and targeting EVs may therefore emerge as a novel approach to delaying ectopic ossification in AS.

## Graphical abstract

Extracellular vesicles (EVs) derived from ligament tissue mediate inter‐tissue communication between ligament and bone in ankylosing spondylitis (AS). EVs carrying interleukin‐17A promote osteogenic differentiation of AS mesenchymal stem cells by altering the cellular cytoskeleton and mechanical signaling. This study provides the first evidence of tissue EVs contributing to pathological ossification in AS, offering potential avenues for targeted therapies.

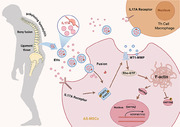

## 1 Introduction

Ankylosing spondylitis (AS) is a chronic inflammatory disorder characterized by a distinct predilection for the sacroiliac, spinal, and hip joints,^[^ ^1^ ^]^ with chronic inflammation and pathological fusion of the spine being a particularly distinctive feature. In the early stages of the disease, inflammatory pain predominates, while in the progressive phase, the spine undergoes a gradual transformation to attain a “bamboo‐like” morphology, resulting in progressive loss of functional mobility and eventually culminating in spinal ankylosing and deformity, necessitating surgical intervention as the sole method of treatment.^[^ ^2^ ^]^

Current pharmacotherapeutic interventions for AS primarily focus on symptom control. Non‐steroidal anti‐inflammatory drugs (NSAIDs), tumor necrosis factor‐alpha (TNF‐α) inhibitors, interleukin‐17 (IL‐17) inhibitors, and more recently, Janus kinase (JAK) inhibitors are regarded as the mainstay drugs in the early therapeutic “golden window” in AS.^[^ ^3^ ^]^ However, despite their effectiveness in controlling inflammation, alleviating symptoms, and slowing disease progression, the majority of patients do not receive satisfactory relief with regard to pathological ossification after treatment.^[^ ^4^ ^]^ Moreover, details of the underlying mechanisms of pathological ossification in AS remain unclear, and the intricate interplay between pathological new bone formation and inflammation remains a critical unresolved puzzle in this context. Undoubtedly, TNF is not the sole factor implicated in driving pathological osteogenesis.^[^ ^5^ ^]^ Whereas both inflammation and mechanical stress have been reported to activate signaling pathways, such as the Hippo, Hedgehog, bone morphogenetic protein (BMP), and Wnt signaling pathways, to contribute to spinal fusion in patients with AS.^[^ ^6^ ^]^ However, a precise understanding of the cellular and molecular mechanisms involved, as well as of whether pro‐inflammatory cytokines coordinate inflammation‐driven bone remodeling, remains elusive.

Mesenchymal stem cells (MSCs) and osteoprogenitor cells are considered responsive entities committed to osteogenesis within the microenvironment conducive to bone formation.^[^ ^7^ ^]^ Notably, both differentiated and recruited MSCs are believed to contribute to pathological ossification,^[^ ^8^ ^]^ but despite some evidence regarding the potential origins of these cells, considerable controversy remains regarding their migration to new bone formation sites and the mechanisms through which they affect pathological ossification. Extracellular vesicles (EVs) serve as crucial communication mediators between cells, tissues, and organs, and play crucial roles in several biological processes. Tissue‐derived EVs are nanoscale vesicles that are present in the tissue interstitium and are secreted by various cells. They are known to transport a diverse array of signaling molecules, including proteins, mRNA, microRNA, and lipids, and participate in numerous biological processes across almost all organisms.^[^ ^9^ ^]^ In AS, immunological synapse vesicle transfer mediates cell‐to‐cell signaling in T‐cell‐mediated diseases.^[^ ^10^ ^]^ Aberrant expression of plasma‐derived EV miRNA in patients may contribute to pathogenic mechanisms in AS by disrupting the homeostasis of effector T cells and Treg cells.^[^ ^11^ ^]^ Ligament sites serve as focal regions for pathological ossification in AS and generate various bioactive mediators that communicate synergistically with peripheral organs and cells. However, the molecular mechanisms affecting EVs derived from AS ligament and the nature of their involvement in the pathogenesis of ossification remain unclear.

In our previous study, we observed immune hyperactivation in the ligament attachments of spinal joints in patients with AS. Specifically, RNA sequencing (RNA‐seq) data and bioinformatics target prediction and validation were utilized to elucidate the activation of inflammatory processes, upregulation of osteogenic markers, and their association with helper T cells (Th cells) and macrophages in ligament tissue of patients with AS. Utilizing Olink technology for quantitative protein analysis of AS ligament tissue EVs (LTEVs), we demonstrated the enrichment of interleukin‐17A (IL‐17A) in AS‐LTEVs compared to that in EVs from healthy donor (HD) ligament tissue. AS‐LTEVs and their IL‐17A cargo have been demonstrated to promote pathological ossification and disease progression by altering the cytoskeleton and mechanical signaling in stem cells. These findings underscore the need to decipher the interplay between EVs and pathological ossification in AS.

## 2 Results

### 2.1 Pathological Ossification and Inflammatory Activation in Uncalcified Ligaments from Patients with AS

**Figure 1:**
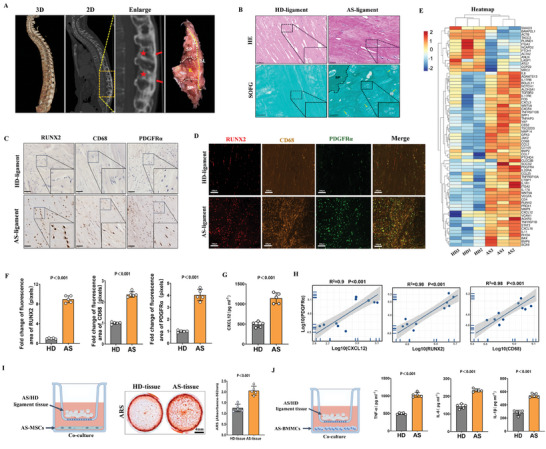
Pathological ossification and inflammatory activation in non‐ossified ligaments from patients with AS. A). µCT images show the 3D reconstruction and spinal ligament tissue from patients with AS. The red pentagram indicates uncalcified ligament. B). SOFG and HE staining of the AS patient ligament sections showing osteogenic differentiation and disorganized arrangement of collagen fibers. C,D). Representative immunohistochemistry and immunofluorescence staining images showing over‐expression of RUNX2, CD68, and PDGFRα in AS spinal ligament tissue. E). Heatmap of differentially upregulated genes, regulation of osteoblast differentiation (GO:0045667), and cytokine‐mediated signaling pathway (GO:0019221). F). Quantitative analysis of the immunofluorescence area occupied by stained regions in the different groups in (D). G). Concentration of CXCL12 in AS ligament tissue measured using ELISA. H). Pearson's correlation analysis of PDGFRα expression and the expression of markers of AS‐related osteoblast differentiation (RUNX2 and CXCL12) and inflammation (CD68) based on the ELISA and immunofluorescence staining results. I). Left: experimental scheme of AS or HD ligament tissue co‐culture with AS‐MSCs. Middle: ARS staining of AS‐MSCs after 21 days of osteogenic induction. Right: quantitative analysis of the ARS staining (absorption, 562 nm). n = 5 per group. J). Experimental scheme of AS or HD ligament tissue co‐culture with AS‐BMMCs. Concentration of inflammation factors after co‐culturing AS or HD ligament tissue with AS‐BMMCs measured using ELISA. IL, interspinous ligament; SL, supraspinous ligament; SP, spinous process; HE, hematoxylin‐eosin staining; SOFG, Safranin O–Fast Green staining.

To investigate the inflammatory hyperactivation in the ligament, spinal ligament tissue was collected from patients with AS and age‐ and sex‐matched controls who had undergone osteotomy surgery (**Figure** **1**A). All tissues used in the experiments were non‐calcified. Safranin O/Fast Green (SOFG) and hematoxylin‐eosin (HE) staining showed that spinal ligament from patients with AS exhibited osteogenic differentiation and a disorganized arrangement of collagen fibers (Figure 1B). Immunofluorescence and immunohistochemical analyses were used to investigate the localization and expression of osteogenic proteins and inflammatory markers in AS ligament tissue and demonstrated that Runt‐related transcription factor 2 (RUNX2), CD68, and platelet‐derived growth factor receptor alpha (PDGFRα) were aberrantly upregulated in AS patient tissue (Figure 1C,D,F). RNA‐seq analyses of the differentially expressed genes in the tissue showed significant enrichment of osteogenic and inflammation‐related Gene Ontology (GO) biological process terms, including “regulation of osteoblast differentiation” (GO:0045667) and “cytokine‐mediated signaling pathway” (GO:0019221) (Figure 1E). Spinal ligament tissue level of C‐X‐C motif chemokine 12 (CXCL12), a stem cell homing factor, was also upregulated in tissue from patients with AS compared to that from non‐AS patients (Figure 1G). Furthermore, the level of PDGFRα was positively correlated with those of markers for AS‐related osteoblast differentiation (RUNX2 and CXCL12) and inflammation (CD68) (Figure 1H). These results indicate that inflammatory processes as well as osteogenic differentiation of MSCs are activated in AS ligament.

MSCs and bone marrow mononuclear cells (BMMCs) are crucial for osteogenesis in ligaments in AS; therefore, we further investigated the possible effects of AS ligament changes on MSCs and BMMCs.^[^ ^12^ ^]^ Both MSCs and BMMCs were obtained from bone marrow tissue isolated from bone sections via centrifugation; the results of analyses to identify the markers of MSCs and their growth curve in the culture medium are shown in Figure S1 (Supporting Information). Subsequently, we homogenized the ligament tissue and co‐cultured it with these cells. As evidenced by Alizarin Red S (ARS) staining, matrix mineralization of AS‐MSCs was enhanced by AS ligament tissue treatment (Figure 1I). Additionally, enzyme‐linked immunosorbent assay (ELISA) results revealed that AS ligament tissue induced increased secretion of the pro‐inflammatory cytokines TNF‐α, IL‐6, and IL‐1β by BMMCs (Figure 1J). These results indicate that AS ligament tissue can induce the osteogenic differentiation of MSCs and stimulate BMMCs to secrete inflammatory factors. Collectively, the histological experiments and RNA‐seq analyses demonstrated that both inflammatory activation and osteogenic differentiation of MSCs occur within ligaments during AS progression. This interesting result piqued our curiosity about how the disease affects ligament tissue behavior, signaling pathways, and the overall ossification response.

### 2.2 Isolation and Characterization of EVs from AS Ligament Tissues

**Figure 2:**
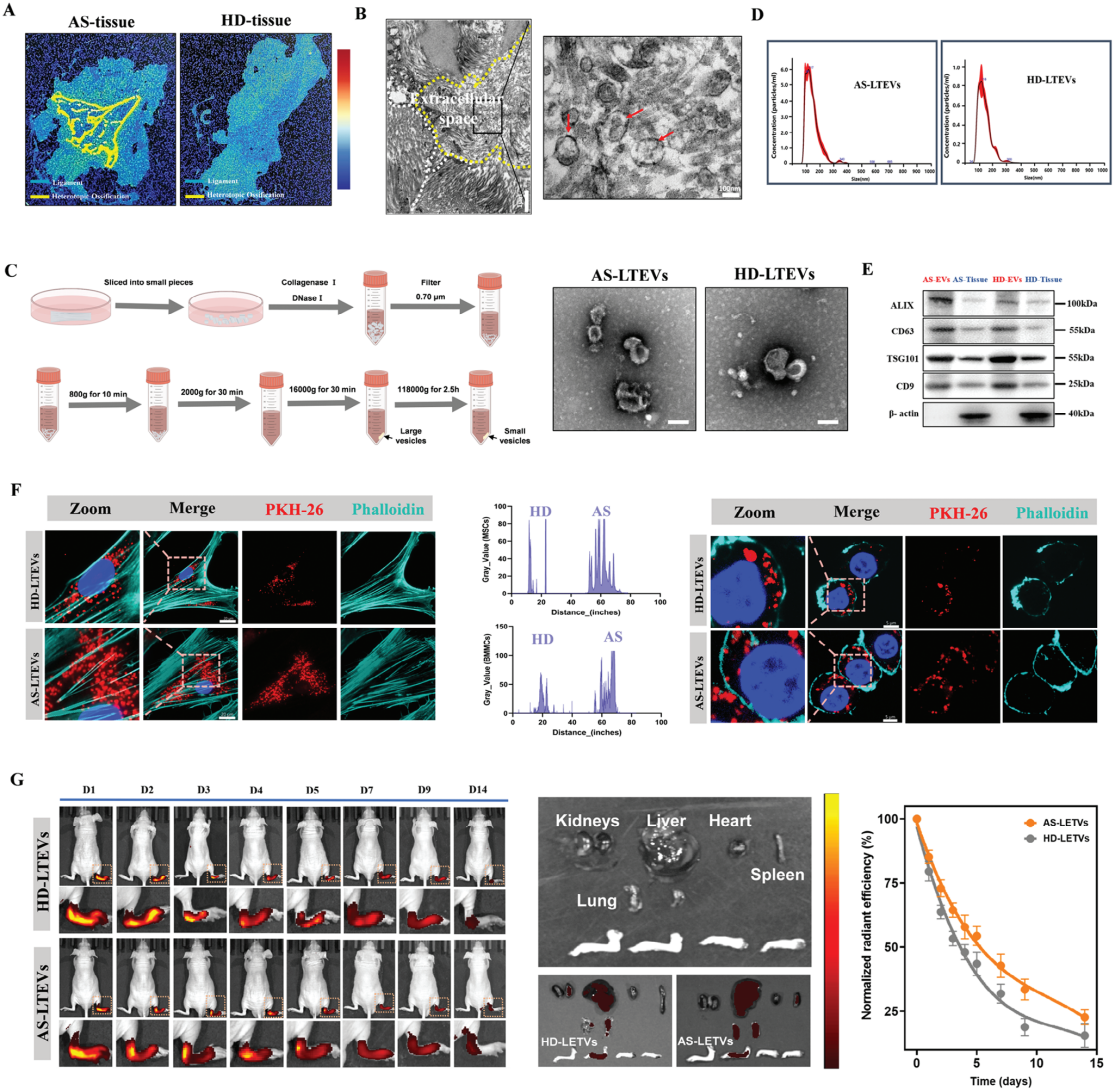
Isolation and characterization of extracellular vesicles from AS ligament tissue. A). Illustration of human spinal ligament tissue collection. Yellow regions indicate pathological new bone. B). TEM was utilized for the examination of extracellular vesicles within the interstitial spaces in spinal ligament tissue. C). Experiment diagram of extracellular vesicle isolation procedure by sequential centrifugation from ligament tissue sections. The right panel shows typical TEM images of EVs from two types of tissues. D). Representative images of NTA of isolated AS‐ and non‐AS LTEVs. E). Western blotting analysis of EV markers (Alix, CD63, TSG101, CD9). F). Uptake of PKH26‐labeled EVs by MSCs and BMMCs. Scale bar, 20 and 5 µm. G). Representative IVIS images of nude mouse over 14 days after single intra‐ankle joint injection of PKH26‐labeled EVs. Quantitative analysis of normalized time‐course fluorescent radiant efficiency in nude mouse ankle joints over 14 days (n = 3 per group). TEM, transmission electron microscopy; NTA, nanoparticle tracking analysis.

In order to investigate how intercellular communication and interactions in ligaments are affected in AS, we used the method recommended by the Minimal Information for Studies of Extracellular Vesicles (MISEV) guidelines for the characterization and functional studies of EVs.^[^ ^13^ ^]^ AS ligament tissue was extracted from non‐calcified regions surrounding ectopic ossification in ligaments (**Figure** **2**A), and transmission electron microscopy (TEM) analysis revealed the presence of vesicles in spinal ligament. Specifically, vesicular structures of various sizes but morphologies similar to those of EVs were observed in spinal fibroblast spaces (Figure 2B).

TEM analysis revealed that the LTEVs had a typical EV morphology with a cup‐shaped or vesicular structure (Figure 2C, right). To further study these vesicular structures, the isolated LTEVs were identified based on size using nanoparticle tracking analysis (NTA), which showed a mean LTEV size of 140.4 ± 46.7 and 141.5 ± 49.8 nm (Figure 2D). The NTA and TEM results indicated no significant differences in the size and morphology between the two types of LTEVs. Furthermore, western blotting analysis confirmed that the collected LTEVs expressed several EV marker proteins, including Alix, CD63, CD9, and TSG101 (Figure 2E). To examine whether the collected EVs could be taken up by other cells, we performed an in vitro experiment using PKH26‐labeled EVs. The results showed strong PKH26 dye signal in the cytoplasm of AS‐MSCs and AS‐BMMCs (Figure 2F). In vivo near‐infrared fluorescence imaging analysis of the biodistribution and biosafety of the EVs revealed maximal fluorescence signals in the ankle joints and paws of healthy mice after PKH26‐EV injection (Figure 2G). Representative images of HE staining of major organs (heart, liver, spleen, lungs, and kidneys) after 4‐week serial injections (once weekly) of HD‐LTEVs and AS‐LTEVs are shown in Figure S2 (Supporting Information). These results conclusively illustrate that LTEVs actively secreted by ligament tissue can mediate inter‐tissue communication between the ligament and other cells.

### 2.3 AS‐LTEVs Mediated Inter‐Tissue Communication and Osteogenic Differentiation between Ligament Tissues and MSCs

Communication between ligaments and cells has been demonstrated in preceding experiments. To determine whether AS‐LTEVs can also induce the osteogenic differentiation of MSCs like AS ligament tissue, AS‐MSCs were co‐cultured with LTEVs to detection of optimal concentration for EV function (Figure S3, Supporting Information).

**Figure 3:**
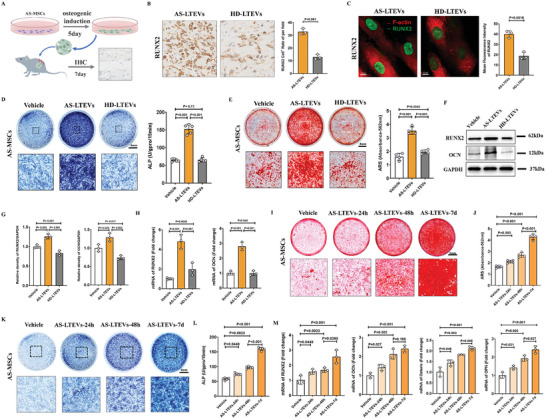
AS‐LTEVs mediated osteogenic differentiation and communication between ligament tissue and MSCs. A). Schematic of the in vivo osteogenesis assay. AS‐MSCs and AS‐ or HD‐LTEVs were transplanted into nude mice. B). Left: immunohistochemical staining to compare the osteogenic effects of AS‐ and HD‐LTEVs on AS‐MSCs in vivo. Scale bar: 20 µm. Right: quantitative analysis of RUNX2‐positive cells in the in vivo osteogenesis assay. n = 3 per group. C). Immunofluorescence staining for RUNX2 in AS‐MSCs treated with AS‐ or HD‐LTEVs for 48 h. Scale bar: 20 µm, n = 3 per group. D). ALP staining and quantitative analysis of AS‐MSCs after treatment with PBS, AS‐LTEVs, or HD‐LTEVs for 48 h with 7 days osteogenic induction. n = 5 per group. E). ARS staining and quantitative analysis of AS‐MSCs after treatment with PBS, AS‐LTEVs, or HD‐LTEVs for 48 h with 21 days of osteogenic induction. n = 5 per group. F,G). Western blotting and quantitative analysis of osteogenesis transcription factors in AS‐MSCs after treatment with PBS, AS‐LTEVs, or HD‐LTEVs for 48 h. n = 3 per group. H). Relative expression of mRNA of key signaling transcription factors involved in osteogenesis in AS‐MSCs determined by RT‐qPCR after co‐incubation with each LTEVs for 48 h. n = 3 per group. I–L). The effects of AS‐LTEVs on AS‐MSCs were assessed at three time points: 24, and 48, and 7 days, using ARS and ALP staining. n = 4 per each group. M). RT‐qPCR analysis of osteogenic markers (RUNX2/OCN/Osterix/OPN) of AS‐LTEVs on AS‐MSCs at 24, and48 h, and 7 days. ALP, alkaline phosphatase; ARS, alizarin red S.

To verify the effectiveness of LTEVs in promoting osteogenesis in vivo, we transplanted AS‐MSCs with LTEVs into nude mice and then performed used an immunohistochemical assay (**Figure** **3**A) to show that a much higher number of RUNX2+ cells was injected with LTEVs in the AS group (Figure 3B; Figure S4A, Supporting Information). Immunofluorescence staining of AS‐LTEVs co‐cultured with AS‐MSCs in vitro demonstrated that AS‐LTEV intervention increased the expression of RUNX2 in AS‐MSCs (Figure 3C; Figure S4B, Supporting Information). Assessment of alkaline phosphatase (ALP) activity (Figure 3D), ARS staining (Figure 3E), western blotting analysis (Figure 3F,G), and RT‐qPCR analysis (Figure 3H) of osteogenic master genes confirmed the positive impact of AS‐LTEVs on the osteogenic differentiation of MSCs. Notably, AS‐LTEVs significantly enhanced osteogenic differentiation as early as between 24 and 48 h, and this effect persisted even at 7 days after induction, indicating a sustained effect of LTEV uptake and activity (Figure 3I–M). When ligament cells obtained by treating ligament tissue collagenase I was co‐cultured with LTEVs, we observed that AS‐LTEVs can also promote the osteogenesis of AS ligament fibroblasts (Figure S5, Supporting Information). These results demonstrate that AS‐LTEVs can promote the osteogenic differentiation of both MSCs and ligament cells.

### 2.4 Proteomic Analysis of AS‐LTEVs using Olink Technology Revealed Elevated Expression of IL‐17A

EVs serve as carriers that transport bioactive components into target cells. The preceding experiments suggested that AS‐LTEVs contain bioactive components that positively regulate bone formation. Proteins, as the main cargo of EVs, play a crucial role in regulating EV‐mediated intercellular communication in numerous biological processes.

**Figure 4:**
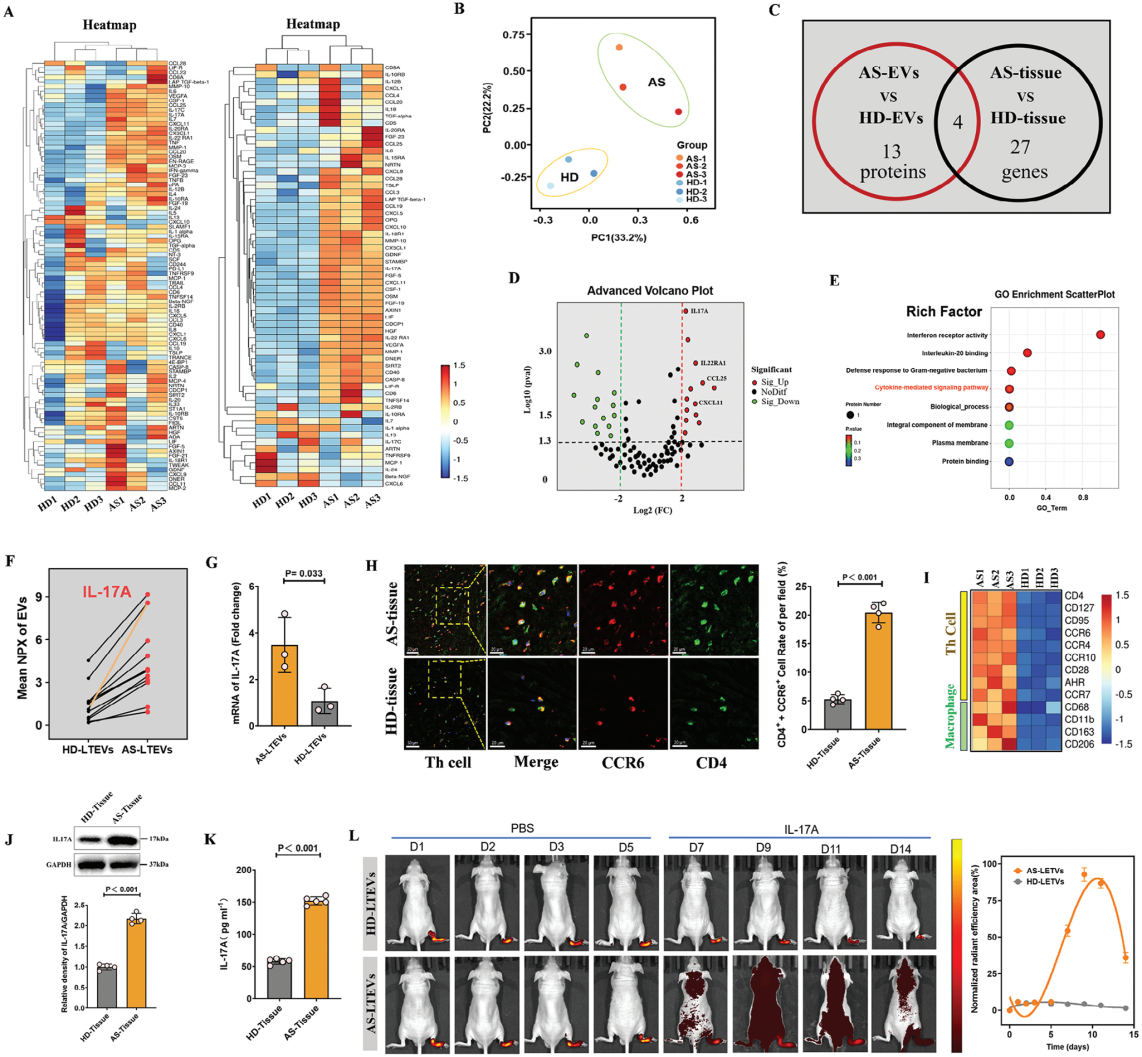
Proteomic analysis of AS‐LTEVs using Olink technology reveals elevated expression of IL‐17A. A). Heatmap showing protein expression in AS‐ and HD‐LTEVs. Different colors represent different levels of protein expression, with blue to red representing low to high levels of expression. B). Olink PCA analysis of HD‐LTEVs and AS‐LTEVs. C). RNA‐seq of the ligaments revealed 27 upregulated genes with an intersection of four upregulated proteins found in the Olink proteomics dataset. D). Volcano plot of upregulated and downregulated proteins in the HD‐ and AS‐LTEV groups. E). The top 8 significant pathways were enriched from the 92 critical proteins in GO enrichment analysis. F). Expression tendency of 13 upregulated proteins between AS‐LTEVs and HD‐LTEVs using Olink proteomics analysis G). RT‐qPCR analysis of IL‐17A expression in EVs. H). Immunofluorescent staining and quantitative analysis of CD4‐ and CCR6‐positive cells in AS and HD spinal ligament. I). Heatmap of differentially upregulated genes of Th cell and macrophage markers in HD and AS spinal ligament. J). Western blotting and quantitative analysis of IL‐17A in AS or HD spinal ligament. K). The concentration of IL‐17A in ligament tissue from HDs and patients with AS was measured using ELISA. L). Representative IVIS images of nude mice over 14 days demonstrating the effect of IL‐17A injection on the in vivo distribution of EVs. Quantitative analysis of normalized time‐course fluorescent radiant efficiency within nude mouse ankle joints over 14 days (n = 3 per group).

To identify the AS‐LTEV proteins responsible for osteogenic differentiation, AS‐LTEVs and HD‐LTEVs were profiled using Olink proteomics (**Figure** **4**A, left). Ninety‐two LTEV proteins were detected and analyzed; thirteen proteins were found to be upregulated in the AS‐LTEV group compared to in the HD‐LTEV group. Principal component analysis (PCA) of Olink also confirmed that there were differences in the expression profiles of AS‐LTEVs and HD‐LTEVs (Figure 4B). Meanwhile, the differential gene expression analysis of Olink‐related target proteins was performed using ligament RNA‐seq (Figure 4A, right). RNA‐seq of the ligaments revealed 27 upregulated genes with an intersection of four upregulated proteins found in the Olink proteomics dataset (Figure 4C). Volcano plot analysis showed that IL‐17A, IL‐22RA1, CCL25, and CXCL11 levels were increased in both AS‐LTEVs and AS ligament tissue (Figure 4D), and “chemokine‐mediated signaling pathway” and “interleukin‐20 binding” terms were identified based on GO enrichment analysis (Figure 4E). To further confirm the proteins most likely responsible for pathological ossification in AS ligaments, the 13 upregulated proteins were analyzed for trend variations between subjects with and without AS. Notably, IL‐17A exhibited a more differential upregulation trend (Figure 4F), and RT‐qPCR results also confirmed the upregulation of IL‐17A expression in AS‐LTEVs (Figure 4G).

Immunofluorescence staining was performed to confirm the presence of conditions conducive for IL‐17A production in AS ligament tissue and the expression of Th cell markers. The results showed CCR6 and CD4 co‐staining in AS ligament, and both of these are commonly used markers of Th cells in ligament tissue (Figure 4H). RNA‐seq analysis of ligament tissue also showed that Th17/22 cell and macrophage cell‐specific biomarker levels were upregulated (Figure 4I). ELISA and western blotting analyses confirmed that the level of IL‐17A in spinal ligament tissue was higher in patients with AS than in non‐AS patients (Figure 4J,K). Finally, IVIS imaging over 14 days demonstrated that IL‐17A injection could accelerate the biodistribution of AS‐LTEVs in nude mice (Figure 4L). These results indicate that the overexpression of IL‐17A in AS‐LTEVs could play a key role in the progression of pathological ossification in AS.

### 2.5 AS‐LTEVs Promote Pathological Ossification by Activating the STAT3/MMP14/Hippo Signaling Pathway

**Figure 5:**
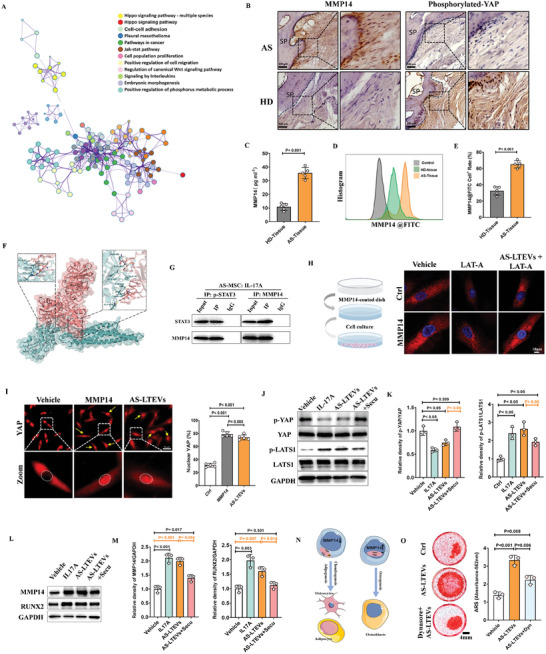
AS‐LTEVs promote pathological ossification by activating the STAT3/MMP14/Hippo signaling pathway. A). Pathway enrichment analysis of differentially expressed genes in spinal ligaments from patients with AS. B). Immunohistochemical staining of phosphorylated YAP (S127) and MMP14 in entheseal tissues. Scale bar: 100 µm. C). The concentration of MMP14 in ligament tissue from HDs and patients with AS was measured using ELISA. D,E). Representative flow cytometry analysis of MMP14‐positive cells in HD or AS spinal ligament. F,G). Protein docking analysis and co‐immunoprecipitation were used to evaluate the associativity of phosphorylated STAT3 and MMP14 with the osteogenic differentiation of AS‐MSCs. H). Immunofluorescence image of actin polymerization (phalloidin, red) and nuclei (DAPI, blue) in AS‐MSCs cultured on MMP14‐coated dishes with or without LAT‐A administration for 12 h. Scale bar: 10 µm. I). Immunofluorescence imaging and analysis of nuclear translocation of YAP in AS‐MSCs treated with AS‐LTEVs or plated on MMP14 for 6 h. Scale bar: 50 µm. n = 5 per group. J,K) Western blotting analysis of Hippo pathway‐related proteins in AS‐MSCs after co‐incubation with each group for 2 days. L,M). Western blotting and quantitative analysis of MMP14 and osteogenic markers. N). Influence of MMP14 expression on trilineage differentiation of AS‐MSCs. O). AS‐LTEVs enhanced AS‐MSC osteogenesis and migration, and this enhancement was inhibited by secukinumab (an IL‐17A inhibitor). LAT‐A, latrunculin A; YAP, Yes‐associated protein.

To explore the downstream signaling pathways of IL‐17A, pathway enrichment analysis was conducted and revealed a significant enrichment of genes related to the Hippo signaling pathway, extracellular matrix (ECM) organization, and positive regulation of protein phosphorylation (**Figure** **5**A; Figure S6, Supporting Information). IL‐17A can activate the JAK/signal transducer and activator of the transcription (STAT) pathway by inducing rapid tyrosine phosphorylation of STAT 1, 2, 3, and 4.^[^ ^14^ ^]^ Our results demonstrated that AS‐LTEVs can also activate the JAK‐STAT3 pathway (Figure S7A, Supporting Information). Matrix metalloproteinase (MMP) family proteins are major players in ECM organization, and RNA‐seq analysis revealed the upregulation of *MMP14* gene expression in AS patient ligament (Figure 1E). Yes‐associated protein (YAP) is the core effector of the Hippo signaling pathway, and its phosphorylation negatively regulates osteogenesis.^[^ ^15^ ^]^

We used immunohistochemical staining to validate the aberrant expression of YAP and MMP14 in AS ligament pathological ossification and observed that phosphorylated YAP and MMP14 levels in the spinal ligament tissue of patients with AS were inversely associated (Figure 5B), and ELISA results showed a significant increase in MMP14 expression in AS ligament tissue (Figure 5C). Furthermore, flow cytometry analysis revealed that the AS‐ligament had more MMP14‐positive cells than HD‐ligament (Figure 5D,E). These results suggest that there is a correlation between the activation of the Hippo and JAK/STAT3 pathways and that this correlation may mediate pathological ossification by affecting the expression of MMP14. To further validate the correlation between the JAK/STAT3 pathway and MMP14, protein docking analysis was used to assess the possible interaction between MMP14 and phosphorylated‐STAT3 (Figure S8A, Supporting Information). ColabFold was used to model the protein interaction structures and conduct a confidence analysis (Figure S8, Supporting Information). Finally, of the five predicted models, the model with the highest confidence score was used to visualize the protein‐protein interaction (Figure 5F; Figure S8F, Supporting Information). Co‐immunoprecipitation experiments indicated that phosphorylated‐STAT3 can interact with MMP14 (Figure S7B, Supporting Information) and that this interaction is enhanced in the presence of IL‐17A (Figure 5G).

The Rho family GTPase‐effector pathway is a major regulator of stress fiber formation, and MMP14 and AS‐LTEVs were found to activate the Rho/Rho‐kinase signaling pathway (Figure S7C, Supporting Information). Stress fiber formation is a major contributor to cytoskeletal modification and MSC differentiation, whereas actin cytoskeleton aggregation and disassembly can affect mechanical signals, which in turn can activate the Hippo signaling pathway.^[^ ^16^ ^]^ To confirm the activating effect of MMP14 on the Hippo signaling pathway, cells were plated on MMP14‐coated dishes to assess actin polymerization (Figure 5H, left).

We observed that MMP14 could promote actin cytoskeleton polymerization in AS‐MSCs and that AS‐LTEVs could reverse the actin cytoskeleton disassembly in AS‐MSCs when latrunculin A (LAT‐A), a cellular actin polymerization inhibitor, was administered (Figure 5H, right). Furthermore, when cells were treated with MMP14 or AS‐LTEVs, the nuclear translocation of YAP increased (Figure 5I). Western blotting analysis showed that the level of phosphorylated large tumor suppressor kinase 1 (LATS1) increased and that of phosphorylated YAP decreased in AS‐MSCs after AS‐LTEV treatment (Figure 5J,K). These findings indicate that AS‐LTEVs contribute to actin cytoskeleton polymerization, activation of the Hippo signaling pathway, and the subsequent nuclear translocation of YAP.

Western blotting analysis also revealed that MMP14 upregulation under the influence of AS‐LTEVs and the enclosed IL‐17A could stimulate the expression of RUNX2 during osteogenic differentiation of AS‐MSCs (Figure 5L,M). MSCs differentiate into osteoblasts when MMP14 level is elevated, whereas reduction in MMP14 level promotes their differentiation into chondrocytes or adipocytes (Figure 5N; Figure S9A,B, Supporting Information). As expected, AS‐LTEVs induction significantly increased matrix mineralization, and inhibiting the endocytosis of AS‐LTEVs could reduce matrix mineralization in AS‐MSCs (Figure 5O). Moreover, AS‐LTEVs could promote the migration of MSCs, potentially facilitating their attachment to ligament sites, and inhibiting the endocytosis of AS‐LTEVs could prevent the migration of AS‐MSCs (Figure S9C, Supporting Information). These results suggest that AS‐LTEVs facilitate AS‐MSC osteogenesis by modulating MMP14 to activate the Hippo signaling pathway.

### 2.6 AS‐LTEVs Facilitate Inflammation Activation and Pathological Ossification by Delivering IL‐17A In Vivo

**Figure 6:**
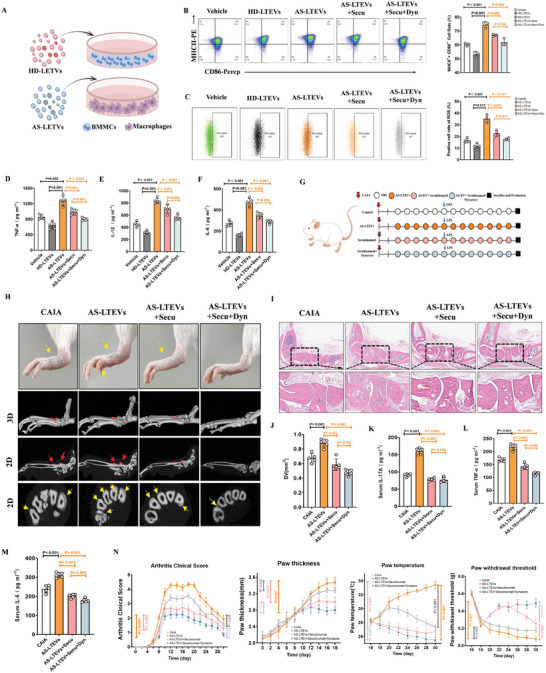
AS‐LTEVs facilitate inflammation activation by delivering IL‐17A in vivo. A). Diagrammatic representation of experimental strategies for macrophage polarization. B). Representative flow cytometry results of M1 macrophages after treatment with PBS, HD‐LTEVs, AS‐LTEVs, AS‐LTEVs with secukinumab, AS‐LTEVs with secukinumab, and Dynasore. Percentages indicate the proportion of M1 macrophages. n = 3 per group. C). Representative flow cytometry results of DCFH‐DA after treatment with PBS, HD‐LTEVs, AS‐LTEVs, AS‐LTEVs with secukinumab, AS‐LTEVs with secukinumab, and Dynasore. The right panel shows the quantitative analysis of the positive cell rate of ROS in the different groups. n = 4 per group. D–F). ELISA‐based measurement of TNF‐α, IL‐10, and IL‐1β levels in supernatants prepared from the culture medium after each of the different treatments in (B). n = 5 per group. G). Schematic diagram of the experimental schedule for the CAIA mouse model. H). µCT scans showing pathological new bone formation in hind paws in different CAIA model mice groups. I). HE staining of CAIA model mice paws and ankle joints sections showing articular destruction and inflammatory cell infiltration. J). Quantitative analysis of structural parameters of new bone by µCT analysis. n = 5 per group. K–M). Concentration of IL‐17A, TNF‐α, and IL‐6 in the serum of CAIA model mice. N. Therapeutic efficacy of the various substances assessed in terms of changes in the main symptoms of CAIA: arthritis clinical score, paw thickness, paw temperature, and pain‐associated behavior analysis using the von Frey filament test. n = 5 per group. DCFH‐DA, dichlorodihydrofluorescein diacetate; ROS, reactive oxygen species.

As macrophage polarization plays a crucial role in activating inflammation, we hypothesized that AS‐LTEVs would amplify this polarization effect. **Figure** **6**A depicts a schematic of the device and experimental design used for this experiment. After 24 h of stimulation, flow cytometry was used to assess the proportion of M1 macrophages with upregulated CD86 and MHC II expression. The results demonstrated that AS‐LTEVs induced an increase in BMMC macrophage (M1) polarization; conversely, the opposite effects were observed in the HD‐LTEV and other treatment groups (Figure 6B; Figure S10A, Supporting Information). Due to the amplified M1 macrophage polarization, the secretion of pro‐inflammatory factors such as TNF‐α, IL‐1β, and IL‐6 also increased (Figure 6D–F).

Similarly, as the overproduction of reactive oxygen species (ROS) is one of the causes of AS inflammation and macrophage activation, the ROS‐producing ability of AS‐BMMCs induced by AS‐LTEVs was investigated. Flow cytometry analysis revealed that AS‐LTEVs induced ROS overproduction in AS‐BMMCs (Figure 6C; Figure S10B, Supporting Information). These results indicate that AS‐LTEVs could activate the inflammatory response of BMMCs derived from bone marrow.

The collagen antibody‐induced arthritis (CAIA) model is characterized by acute inflammatory responses and pathological ossification of the metatarsophalangeal joint (Figure S11, Supporting Information). To confirm the critical role of AS‐EVs in pathological new bone formation in vivo, AS‐LTEVs with or without an IL‐17A antagonist (secukinumab) and an endocytosis antagonist (Dynasore) were administered to CAIA model mice after the first induction dose (Figure 6G). Following intervention with AS‐LTEVs in CAIA mice, µCT analysis revealed that pathological new bone formation in the hind paws was strengthened. However, combination therapy with secukinumab and Dynasore inhibited the osteogenic effects of AS‐LTEVs (Figure 6H,J). Furthermore, ankle and paw inflammatory swelling was augmented by AS‐LTEVs compared to that in the CAIA group (Figure 6I). ELISA results showed that the serum levels of the inflammation factors TNF‐α, IL‐17A, and IL‐6, were increased by the administration AS‐LTEVs (Figure 6K–M). Meanwhile, arthritis clinical score, paw thickness, paw temperature, and paw withdrawal threshold increased in CAIA model mice in the AS‐LTEV group and decreased in the combination therapy group (Figure 6N). These results strongly suggest that AS‐LTEVs activated inflammation and pathological new bone formation in this AS animal models owing to the presence of IL‐17A within the vesicles.

**Figure 7:**
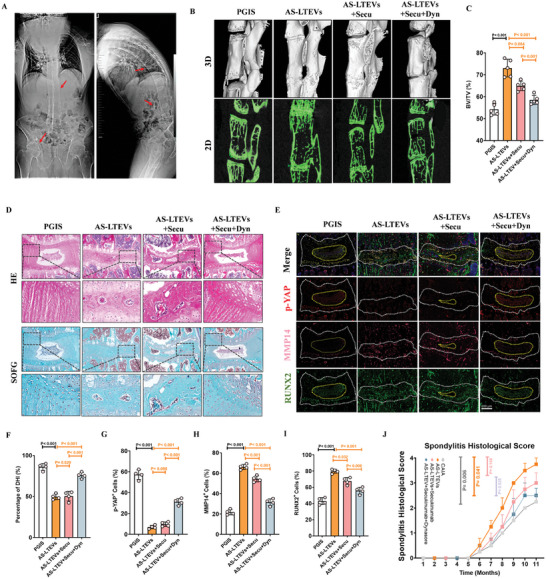
AS‐LTEVs facilitate pathological new bone formation by delivering IL‐17A in vivo. A). Radiological images of patients with AS. Red arrows indicate pathological new bone formation and ankylosis of the hip and spine. B). µCT scans showing pathological new bone formation in PGIS model mouse spine. C). Quantitative analysis of structural parameters of newly formed bone based on µCT analysis (n = 5 per group). D). HE and SOFG staining of new bone from PGIS model mice. E). Representative immunofluorescence staining images showing phosphorylated YAP, MMP14, and RUNX2 in the spine of PGIS model mice. Scale bar: 200 µm (n = 5 per group). F). µCT scanning and HE staining results indicate a markedly reduced DHI because of disc bone formation in PGIS model mice. G–I). Quantitative analysis of phosphorylated‐YAP, MMP14, and RUNX2 in PGIS model mice. J). Therapeutic efficacy of various test substances in the PGIS model in terms of changes in spondylitis histological score.

Spinal ankylosis and hip joint stiffness are the main features of AS due to structural damage and bony fusion in axial joints, including the spinal, sacroiliac, and knee joints (**Figure** **7**A). A proteoglycan‐induced spondylitis (PGIS) mouse model exhibiting both axial and peripheral inflammation was established to observe the pathological changes in spinal ankylosis. µCT analysis showed that spinal ankylosis gradually developed at around 24 weeks after model induction (Figure S12, Supporting Information), and AS‐LTEVs could accelerate the rate of pathological ossification at the 24th week in the PGIS model (Figure 7B). µCT results also revealed an increase in bone volume/ total volume (BV/TV) in the AS‐LTEV group, whereas a decrease was observed in the combination therapy group (Figure 7C). HE and SOFG staining revealed the pathological process of new bone formation at the spinal entheseal and disc site (Figure 7D).

Meanwhile, the disc height index (DHI) was suppressed in the PGIS model, and the inhibition of AS‐LTEV endocytosis restored the DHI suppression caused by pathological ossification (Figure 7F). Consistent with the osteogenic process of AS‐MSCs, immunofluorescence staining revealed that MMP14 and phosphorylated YAP were primarily co‐stained with RUNX2 in the spine of PGIS model mice (Figure 7E). Combination therapy could decrease the expression of MMP14 and promote YAP phosphorylation (Figure 7G–I). The therapeutic efficacies of various substances were assessed in terms of the spondylitis histological score in the PGIS model (Figure 7J). These results indicate that the combination therapy of secukinumab and Dynasore targeting AS‐LTEVs significantly suppressed pathological new bone formation in AS animal models.

## 3 Discussion

**Figure 8:**
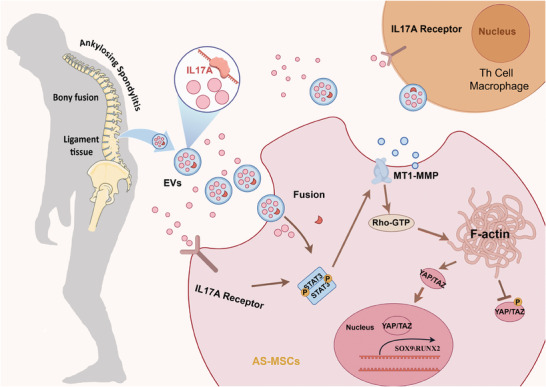
Signaling pathways through which AS‐LTEVs promote AS‐MSCs osteogenic differentiation by inducing alterations in the cellular cytoskeleton and mechanical signaling. Schematic diagram illustrating how EVs derived from ligament tissue and the enclosed interleukin‐17A mediate inter‐tissue communication between ligament tissue and cells. EVs stimulate macrophages to produce inflammatory factors and recruit and stimulate MSCs for osteogenic differentiation. MT1‐MMP, membrane‐type 1 matrix metalloproteinase (also known as MMP14).

The correlation between inflammation and pathological new bone formation has so far not been clarified.^[^ ^6c^ ^]^ Owing to the limited understanding of the molecular mechanisms underlying AS, effective therapeutic strategies involving pharmacotherapy or preventive interventions remain elusive. This leaves surgical intervention as the sole treatment option for patients in advanced stages with severe ankylosing deformities.^[^ ^4^ ^]^ Thus, a better understanding of the mechanism of pathological new bone formation in AS is needed to further develop nonsurgical treatment approaches (**Figure** **8**).

Disease‐related EVs can strongly influence the ability of cells to proliferate and differentiate.^[^ ^17^ ^]^ Both EV‐enclosed proteins and RNA are vital players in numerous biological and pathological processes. EVs derived from peripheral blood have been reported to contribute to immune cell activation in different situations and to affect disease activity and severity in AS.^[^ ^11^, ^18^ ^]^ In addition, EVs derived from spinal ligament cells contribute to the ossification of the posterior longitudinal ligament by promoting osteogenic differentiation of MSCs and ligaments.^[^ ^19^ ^]^ Since EVs contribute to various inflammatory and ossification conditions and AS is a chronic inflammatory disease, it is reasonable to speculate that AS‐EVs may contribute to the process of pathological new bone formation in AS. In the current study, we were especially intrigued by the tissue immunohistochemistry and RNA‐seq analyses results. Infiltration of osteogenic cells and expression of inflammatory cytokines in ligament tissue samples collected from patients with AS indicated that regions of potential pathological new bone are inflamed, which is consistent with previous studies.^[^ ^20^ ^]^ EVs derived from uncalcified ligament tissue in AS were extracted. We discerned a pronounced augmentation of these vesicles in both osteogenic processes and inflammatory responses. Remarkably, upon administration to spine and ankle joints of mice, these AS‐LTEVs demonstrated a remarkable capacity to amplify pathological ossification. These findings strongly suggest that AS‐LTEV‐mediated AS‐MSC osteogenic activity plays a critical role in pathological new bone formation in AS.

The critical question we aimed to answer through this study was how AS‐LTEVs are responsible for the development of pathological new bone formation at ligament entheseal sites in AS. We used Olink proteomics combined with tissue RNA‐seq analysis to show for the first time that IL‐17A is upregulated in LTEVs from patients with AS. Furthermore, we confirmed that AS‐LTEVs contributed to potential pathological new bone formation in vitro and in the CAIA/PGIS models. Intriguingly, we found that the arthritis clinical score decreased with the administration of an IL‐17A antagonist in CAIA mice, indicating that AS‐LTEV‐enclosed IL‐17A may play a role in maintaining the inflammatory microenvironment. Furthermore, this enhanced osteogenic activity and serum IL‐17A concentration could be effectively suppressed using inhibitors targeting IL‐17A, indicating that IL‐17A delivered from AS‐LTEVs plays a crucial role in the pathological new bone formation in ligament.

Bone formation begins with the convergence of MSCs at the site of future bone formation. During intramembranous ossification, MSCs proliferate and undergo compact condensation, and a subset of these MSCs differentiates into pre‐osteoblasts and then osteoblasts.^[^ ^21^ ^]^ Some recent studies provide strong evidence for a direct and crucial contribution of osteoblasts in direct osteogenesis in AS.^[^ ^7^, ^12^ ^]^ Several studies have also demonstrated pro‐osteoblastogenic effects of IL‐17A. IL‐17A is produced by Th17 cells, the main T cell subset, and can stimulate osteoblast differentiation and migration.^[^ ^22^ ^]^ IL‐17A has been shown to activate MMPs and promote matrix turnover during chronic inflammation, leading to cartilage loss in joint tissue.^[^ ^22^, ^23^ ^]^ MMP14 plays an important role in ECM remodeling and enhancing cell migration.^[^ ^24^ ^]^ Consistently, we found high MMP14 expression in AS ligament tissue, and stimulation of AS‐MSCs with AS‐LTEVs in vitro significantly increased MMP14 expression. The downstream molecular signaling pathways of MMP14 vary in different cells and pathological processes. In this study, we found that aberrant AS‐LTEVs and their cargo IL‐17A could activate mechanical signals through the upregulation of MMP14 in AS‐MSCs. Previous studies have shown that inhibition of actin depolymerization enhances osteoblast differentiation and bone formation in human stromal stem cells. Cytoskeletal modification mediated by Rho GTPases is a major contributor to the differentiation and migration of MSCs.^[^ ^25^ ^]^ Furthermore, the cytoskeleton undergoes differential modifications during osteogenesis and adipogenesis.^[^ ^26^ ^]^ During osteogenic differentiation, actin polymerization increases, leading to the generation of more stress fibers, and actin bundles are clearly visible under a microscope. However, actin polymerization is reduced in adipogenic differentiation.^[^ ^26^, ^27^ ^]^ This is consistent with our finding that upregulation of MMP14 by AS‐LTEVs mediated the promotion of actin polymerization.

Cytoskeletal modification appears to be an early event guiding the differentiation of MSCs.^[^ ^28^ ^]^ Actin polymerization resulted in increased nuclear localization of YAP/TAZ through the activation of the Hippo pathway.^[^ ^16^, ^29^ ^]^ YAP shuttles between the cytoplasm and nucleus in response to substrate stiffness and other environmental cues, and cytoplasmic localization of YAP is associated with softer surrounding ECM and adipogenic conditions, whereas YAP translocation to the nucleus occurs with a stiffer ECM and osteogenic microenvironment.^[^ ^30^ ^]^ RUNX2 being the first YAP interaction partner identified,^[^ ^31^ ^]^ mechanical signals leads to the nuclear translocation of YAP thus promoting the expression of interaction partner genes and resulting in accelerated mineralization in osteoblasts. This is consistent with our experimental finding that activation of the Hippo pathway can be effectively suppressed by inhibitors targeting IL‐17A. Taken together, these results indicate that aberrant AS‐LTEVs play a vital role in influencing the mechanical signals of MMP14, resulting in YAP translocation to the nucleus and subsequent enhancement of pathological new bone formation.

IL‐17A inhibitors such as bimekizumab, secukinumab, and ixekizumab have been used in several clinical trials on the treatment of AS as well as psoriatic arthritis and certain skin diseases.^[^ ^32^ ^]^ However, although pathological ossification is a long‐term, chronic process there has been no long‐term clinical follow‐up assessment of the efficacy of IL‐17A inhibitors in inhibiting ossification. Our study demonstrated that AS‐LTEVs may serve as a way to mediate the release of IL‐17A. Therefore, considering the potential role of EVs in the disease process, they could provide a solution to the challenge of preventing pathological ossification in AS. Monotherapy alone is unlikely to prevent ectopic ossification while controlling inflammation. Hence, combination anti‐inflammatory and anti‐ossification therapies have been adopted.^[^ ^33^ ^]^ Dynasore, an inhibitor of the proteins involved in endocytosis, inhibited the endocytosis of EVs. We found that pathological ectopic ossification and inflammation in the CAIA and PGIS models could be significantly inhibited by combined use of IL‐17A inhibitors and endocytosis inhibitors. Hence, elucidation of the mechanisms underlying pathological ossification and exploration of combination therapies for AS may make it possible to fundamentally alleviate AS.

Nonetheless, this study had several limitations. The collection of AS tissue samples relies on late‐stage patients with extensive spinal fusion, and there may be variations in pathological ossification at ligament sites. In addition, although the PGIS and CAIA models share similarities with AS and are widely used in AS research, they cannot fully replicate its pathophysiology. Therefore, further research to establish a more representative animal model of AS is required. Moreover, while the focus of this study was on the aberrant activation of Hippo in AS‐MSCs, other types of cells and pathways may also be activated, and their role in other pathological processes must also be investigated.

## 4 Conclusion

In summary, we demonstrated that AS‐LTEVs promote pathological new bone formation through IL‐17A‐mediated activation of Hippo pathway signaling. These findings provide novel mechanistic insights into pathological new bone formation and a better understanding of its relationship with mechanical strain, which could lead to the development of novel therapeutic strategies for pathological new bone formation.

## 5 Experimental Section

### Human Tissue

This study conforms to the Declaration of Helsinki and approved by the Ethical Committee of Nanjing Drum Tower Hospital, Affiliated Hospital Medical School, Nanjing University (#2011052). Demographic and clinical data of patients are shown in Tables S1 and S2 (Supporting Information). All patients provided written informed consent. AS patients who were unable to maintain a horizontal gaze and comfortable extension of the sacroiliac and knee joints in a natural upright position met the criteria for deformity correction. Non‐AS patients all had spinal fractures and met the criteria for surgical treatment.

### Homogenization of Ligament Tissue

All procedures were performed at 0–4 °C. The non‐ossified ligament tissue of interest was excised during surgery, and fat and connective tissue were trimmed from the ligament and discarded. The tissue was placed in cold homogenization buffer (20 mm HEPES with inhibitor cocktails [Table S3, Supporting Information]) and diced into small pieces (1 cm cubes) using a knife. Alternatively, the tissue was passed twice through a meat grinder. Thereafter, 3–4 volumes of homogenization buffer per volume of tissue was added and the mixture was transferred into a glass‐Teflon homogenizer. The tissue was homogenized at 500–1500 rpm, allowing 5–10 s per stroke. Wash the homogenization with 20 mm HEPES three times to remove inhibitor cocktails. The resulting tissue homogenate was used for subsequent co‐culture experiments.

### Mice Models

All PGIS mice were derived using 8‐week‐old male BALB/c mice (weight, 18–24 g). PGIS mice were obtained using standard methods as previously described.^[^ ^34^ ^]^ Briefly, 100 mg proteoglycan emulsified in complete Freund's adjuvant (Chondrex, WA, USA) was subcutaneously injected into the back of each mouse. On weeks 3 and 6 from the first immunization, a booster injection of the same doses of antigen emulsified in incomplete Freund's adjuvant (Chondrex, WA, USA) was administered. Induction of CAIA was performed by intravenous injection of 1.5 mg of a 5‐clone cocktail on day 0, followed by intraperitoneal injection of 25–50 µg of LPS on day 3 in susceptible BALB/c mice.^[^ ^35^ ^]^ All animal experiments in this study were approved by the Animal Care and Use Committee of the Affiliated Drum Tower Hospital of Nanjing University Medical School (DWSY‐22022148). The same particle dose (1×10^9^, 100 µl) with in vitro experiments is administered injections once a week in the in vivo experiments to inhibit inflammation and ectopic bone formation in animals. All experimental procedures conducted on mice strictly adhered to the rules and guidelines for the ethical use of animals.

### Cell Culture

Human bone marrow stem cells (MSCs) and bone marrow mononuclear cells (BMMCs) were isolated from 10 patients (5 AS patients and 5 non‐AS patients, Table S1, Supporting Information), purified by using density gradient centrifugation after red cell lysis, and further cultured in Dulbecco's modified Eagle's medium (Gibco, USA) containing 10% fetal bovine serum (Cellmax, China) and 1% penicillin‐streptomycin (Gibco, USA) and cultured in a humidified incubator at 37 °C and 5% CO_2_. MSCs were induced for trilineage differentiation experiments using osteogenic, chondrogenic, and adipogenic differentiation media, respectively.

### Osteogenic Differentiation Culture

MSCs were cultured in a complete medium (a‐MEM with 10% fetal bovine serum and 1% penicillin and streptomycin). After 2 days, the medium was changed, and the MSCs were seeded at a density of 1 × 10^4^ cells per well in a 24‐well plate and cultured for 24 h. Thereafter, the complete medium was replaced with osteogenic differentiation basal medium (Cyagen), and LTEVs (1×10^8^ particles/ml) were added for 24, and 48 h, or 7 days with differentiation basal medium change every 2 days. Osteogenic differentiation was assessed using 1% ARS (Cyagen) staining. Calcified nodules were eluted with 10% cetylpyridinium chloride, and absorbance was measured at 562 nm.

### Isolation and Characterization of Ligament EVs

Thirty patients (20 AS patients and 10 non‐AS patients) osteotomy tissue was enrolled to extract EVs. Non‐ossified ligament tissue was collected from surgical specimens, washed three times with PBS, cleared of attached fat and connective tissue, and then cut into small pieces weighing 0.5–20 g. These were then sliced into small pieces of thickness 3–4 mm on ice, which were then incubated with collagenase I (2 mg ml^−1^; Sigma, USA) and deoxyribonuclease I (40 U/ml; Sigma, USA) for 2 h at 37 °C. Filtration through a 0.70 µm pore‐size filter (Millipore, USA) was used to remove large tissue blocks, and the filtrate was then differentially centrifuged at 800 g for 10 min and 2000 g for 30 min to remove cells and tissue debris. The obtained supernatant was then further centrifuged at 16,000 g for 30 min to collect large EVs; small EVs were collected by ultracentrifugation at 118,000 g for 2.5 h. All centrifugations were performed at 4 °C. Large EV‐ and small EV‐enriched pellets were mixed and resuspended in PBS.^[^ ^36^ ^]^ Ligament‐derived EVs were resuspended in 200 µl of PBS for NTA, TEM, and western blotting or used for co‐culture with MSCs or BMMCs.

### Analysis of LTEVs Distribution

Purified PKH26‐labelled LTEVs (1×10^9^ particles/ml, 100 µl) were administered to CAIA model mice to evaluate biodistribution. The mice were imaged using the IVIS Spectrum system 1–14 days after injection, and the major organs (heart, liver, spleen, lungs, and kidneys) and paws were harvested for in vitro and in vivo imaging for 1–2 s (excitation, 551 nm; emission, 567 nm). Data were analyzed using IVIS Living Image Software).

### Macrophages Polarization

Bone marrow was obtained by centrifugation of bone sections, and single‐cell suspensions were prepared after red cell lysis. Mononuclear cells were cultured in a complete DMEM medium containing 10% fetal bovine serum (Dakewe, China). Mononuclear cells were induced to differentiate into macrophages by adding M‐CSF (25 µg ml^−1^) to complete culture medium. The matured macrophages were seeded and replated, followed by the addition of LPS (50 µg ml^−1^) and IFN‐γ (20 µg ml^−1^) to induce polarization towards M1. After 24 h of stimulation, flow cytometry was used to detect the proportion of M1 macrophages with upregulated CD86 and MHCII expression.

### Flow Cytometry

EVs were added at the time of initiation of macrophage polarization and incubated for 48 h. The following antibodies were used to detect expression of cell surface antigens by flow cytometry analysis as previously described: CD11b‐APC (eBioscience, USA), CD68‐FITC (eBioscience, USA), CD86‐Percp (eBioscience, USA), and MHCII‐PE (eBioscience, USA). BMMCs were blocked and then incubated with antibodies for 30 minutes, washed three times, and resuspended in PBS. Candidate cells were detected using a BD Biosciences Influx cell sorter and analyzed using FlowJo v10.0 software. CD11b and CD68 positive cells were identified as macrophages, and the expression levels of CD86 and MHCII were detected to assess the M1 polarization proportion of BMMCs.

### Intracellular ROS Assay

The reactive oxygen species (ROS) detection kit (Beyotime, China) was used according to the manufacturer's instructions. Briefly, EVs were co‐incubated with macrophage for 48 hours. Macrophages were washed three times with PBS and then incubated with DCFH‐DA. After incubation at 37 °C for 20 min, the cells were observed under a fluorescence microscope, or collected for measurement of DCF fluorescence and mean fluorescence intensity (MFI) using flow cytometry (BD Accuri C6 Plus, BD Bioscience, USA) to assess intracellular ROS levels.

### RNA Sequencing and Data Analysis

Total mRNA was extracted from AS‐ligament tissue or HD‐ligament tissue according to the manufacturer's instructions (Thermo Fisher Scientific, USA). The NanoDrop ND‐1000 (NanoDrop, USA) was used to control the amount and purity of total RNA. The Agilent 2100 (Agilent, USA) was used to test the integrity of RNA, and all samples had an RNA Integrity Number (RIN number) >7.0 (indicating the quality of the samples was appropriate for subsequent mRNA analysis). The purified mRNA was fragmented and reverse‐transcribed to create the final cDNA library in accordance with the protocol for the mRNA‐Seq sample preparation kit (Illumina, San Diego, USA). The cDNA libraries were then sequenced on an Illumina Novaseq 6000 (LC‐Bio Technology Co.) according to the manufacturer's instructions.

The volcano plot, heatmap, and KEGG analysis of differentially expressed genes (DEGs) were generated with OmicStudio (https://www.omicstudio.cn/index) tools with clusters Profiler R package. Heatmaps were generated by uploading the FPKM values for the three groups on the website. The screening criteria for DEGs were |log2FC|≥1 and p<0.05. Functional enrichment analysis, including GO functional annotation and KEGG pathway enrichment analysis, was conducted for DEGs. Gene ontology functional annotation was performed using Metascape^[^ ^37^ ^]^ to functionally annotate genes.

### Olink Proteomics

EVs proteins were measured using an Olink Inflammation panel (Olink Proteomics, Sweden) according to the manufacturer's instructions. Briefly, pairs of oligonucleotide‐labeled antibody probes bind to the target protein. The addition of DNA polymerase results in a DNA polymerization reaction that produces a unique PCR target sequence. Subsequently using a microfluidic real‐time PCR instrument (Signature Q100, LC‐Bio Technology Co.) The data were then quality controlled and normalized using an internal extension control and an inter‐plate control to adjust for intra‐ and inter‐run variation.

### Quantitative Real‐Time PCR (RT‐qPCR)

Total RNA was isolated from MSC and ligament tissue using TRIzol reagent (Invitrogen, USA) and transcribed into cDNA using a PrimeScriptTM RT reagent kit (Vazyme, China) according to the protocols. Quantitative real‐time PCR was performed on the LightCycler480 PCR System (Roche, Switzerland) using a SYBR Green Q‐PCR kit (Vazyme, China). The relative expression levels of each gene were analyzed using the 2−^△△Ct^ method. The forward and reverse primers for each gene are shown in Table S4 (Supporting Information).

### Coating with Purified ECM Molecules

MMP14 and RGD peptides (GCGYGRGDSPG, Genscript) were coated on glass coverslips or Matrigel using protocols as previously described, Briefly, FN and TNC were coated in 0.01% Tween 20‐PBS at 1–2 ug cm^−2^ before saturation with 10 mg ml^−1^ heat inactivated BSA/PBS.

### Enzyme‐Linked Immunosorbent Assay (ELISA)

The following ELISA kits were used according to the manufacturer's protocol: CXCL12 (Solarbio, China), TNF‐α (Solarbio, China), IL‐1β (Solarbio, China), IL‐6 (Solarbio, China), and MMP14 (Thermo Scientific, USA).

### Western Blot

Total protein was extracted from cell and tissue samples using RIPA buffer (Sigma, USA), supplemented with phosphatase inhibitors (Bimake, USA) and protease inhibitors (Proteintech, China). Cytoplasmic and nuclear proteins were separated using a cytoplasmic and nuclear extraction kit (Proteintech, China). The protein concentration was determined using a BCA protein assay kit (Proteintech, China). Standard proteins were separated on 7.5‐12.5% SDS‐PAGE gels and transferred onto polyvinylidene difluoride membranes (Millipore, USA) by electrophoresis. The membranes were blocked with 5% skim milk. Subsequently, primary antibodies were incubated with the membranes overnight at 4 °C. Then, secondary antibodies (Abcam, UK) were added at room temperature for 1 h. Membrane signals were detected using immobilized Western chemiluminescent HRP substrate (Millipore, USA). Quantitative densitometry analysis of protein expression was performed using ImageJ (version 1.80).

### Immunofluorescence Staining

The intestinal pathological tissues fixed in formalin and embedded in paraffin were fixed in 4% paraformaldehyde for 15 min. After washing the sections with PBS, they were treated with Triton X‐100 for 15 min (Solarbio, China) and blocked for 1 hour with 5% goat serum (Proteintech, China). Subsequently, the sections were incubated with primary antibodies for 24 h. On the following day, the sections were treated with Alexa Fluor‐conjugated secondary antibodies for 1 hour, followed by DAPI staining (Beyotime, China). The results were observed under a confocal microscope (Leica, Germany).

### Co‐Immunoprecipitation (Co‐IP)

Treating MSCs with IL‐17A for 48 hours, cells were lysed using cell lysis buffer containing both protease and phosphatase inhibitors to extract total cellular proteins. To validate the interaction between phosphorylated STAT3 and MMP14, the lysates were then incubated overnight at 4 °C with anti‐phosphorylated STAT3 antibody or IgG. Subsequently, protein A/G magnetic beads were added and incubated at 4 °C for 4–6 h. After multiple washes, immunoprecipitated protein complexes were separated, and the interaction between phosphorylated‐STAT3 and MMP14 was identified through western blot analysis.

### Protein Docking Analysis

Soft protein–protein docking was performed between phosphorylated‐STAT3 and MMP14 to investigate the relationships by using HDOCK SERVER (http://hdock.phys.hust.edu.cn).^[^ ^38^ ^]^ The protein structural domains of phosphorylated‐STAT3 and MMP14 were obtained from the Protein Data Bank PDB database (http://www.rcsb.org/)^[^ ^39^ ^]^ and Uniport (https://www.uniprot.org/).^[^ ^40^ ^]^ Pymol (Version 2.6) and Colab (https://colab.research.google.com/github/sokrypton/ColabFold/blob/main/AlphaFold2.ipynb)^[^ ^41^ ^]^ were used to investigate protein‐protein interactions and further visual analysis.

### Histological Staining

Tissues were fixed in formalin buffer, decalcified in 0.5 m EDTA, dehydrated in graded ethanol, embedded in paraffin, and sectioned. The tissue sections were placed on a hot plate and baked at 65 °C for 1 h, followed by dewaxing and hydration according to the aforementioned procedure. Sections were stained with hematoxylin and eosin (HE) or Safranin O/fast green (SOFG) to evaluate general structure and bone formation. Immunohistochemical analysis was performed using specific antibodies on specimens. The number of positive cells in each area was quantified according to the aforementioned regions.

### μ‐CT Analysis

Micro‐CT scanner (mCT80; Scanco Medical AG, Switzerland) was used to analyzed human ligament and mice tissue. 2D and 3D images were reconstructed using Scanco Medical software. Quantification of heterotopic ossification was performed using the parameters bone mineral density/ tissue volume (BV/TV) and bone volume (BV).

### Arthritis Clinical Score

The clinical symptoms of the mice were monitored twice a week. The severity of joint swelling was scored as follows: 0 for no joint swelling, 0.1 for swelling in a single toe joint, 0.5 for mild swelling in the wrist or ankle joint, and 1.0 for severe swelling in the wrist or ankle joint.^[^ ^42^ ^]^ Histological score of the spine was determined on a scale of 1–4: 1 = few infiltrating immune cells, 2 = mild inflammation of the discs or along the vertebrae (0–30% of the discs), 3 = inflammation of the discs and/or along the vertebrae (30–70% of the discs), and 4 = inflammation of 70% of the discs and along the vertebrae.^[^ ^43^ ^]^ Pain‐associated behavior of CAIA‐induced animals was assessed by measuring the mechanically induced pain threshold using a series of von Frey filaments as previously described.^[^ ^44^ ^]^

### Statistical Analysis

Data obtained from experiments in duplicate or triplicate and repeated at least three times were represented as mean SD. For quantitative analysis, a minimum of three biological replicates were analyzed. The normality of the data was assessed using the Shapiro‐Wilk test. The significant differences between two samples were compared by a two‐tailed nonpaired Student's *t*‐test. The multiple‐comparison tests were performed using one‐way analysis of variance (ANOVA). One‐way ANOVA was performed with Levene's test for homogeneity of variance, followed by the Bonferroni post hoc test based on the comparison to be made and the statistical indication of each test. A *p*‐value < 0.05 was considered statistically significant. All the data were summarized using Origin 2019 software (OriginLabs, USA).

## Conflict of Interest

The authors declare no conflict of interest.

## Author Contributions

K.Y.W., J.S.L., C.Y.S., and M.Q. contributed equally to the experimental performing, data acquisition and analysis, and manuscript drafting. M.Q., M.H.C., and H.D.B. contributed to clinical tissue sample collection. K.Y.W., C.Y.S., and M.Q. contributed to data analysis and interpretation. K.Y.W. contributed to electron microscopy experiments. B.P.Q. and Y.Q. contributed to manuscript revision. All authors have read and approved the current version of the manuscript.

## Supporting information



Supporting Information

## Data Availability

The data that support the findings of this study are available on request from the corresponding author. The data are not publicly available due to privacy or ethical restrictions.
