# Supplementary material for: Extracellular Vesicles Derived from Ligament Tissue Transport Interleukin‐17A to Mediate Ligament‐To‐Bone Crosstalk in Ankylosing Spondylitis
Source: Adv Sci (Weinh). 2024 Sep 23;11(46):2406876. doi: 10.1002/advs.202406876 (PMC11633500; doi:10.1002/advs.202406876)
Supplement: Supplementary file 1 — Supporting Information [file ADVS-11-2406876-s001.docx]

**Supporting Information for**

**Extracellular vesicles derived from ligament tissue transport interleukin-17A to mediate ligament-to-bone crosstalk in ankylosing spondylitis**

Kaiyang Wang et al.

Corresponding author: Bang-ping Qian, qianbangping@nju.edu.cn

**This PDF file includes:**

Figs. S1 to S12

Tables S1 and S6

Figure-S1


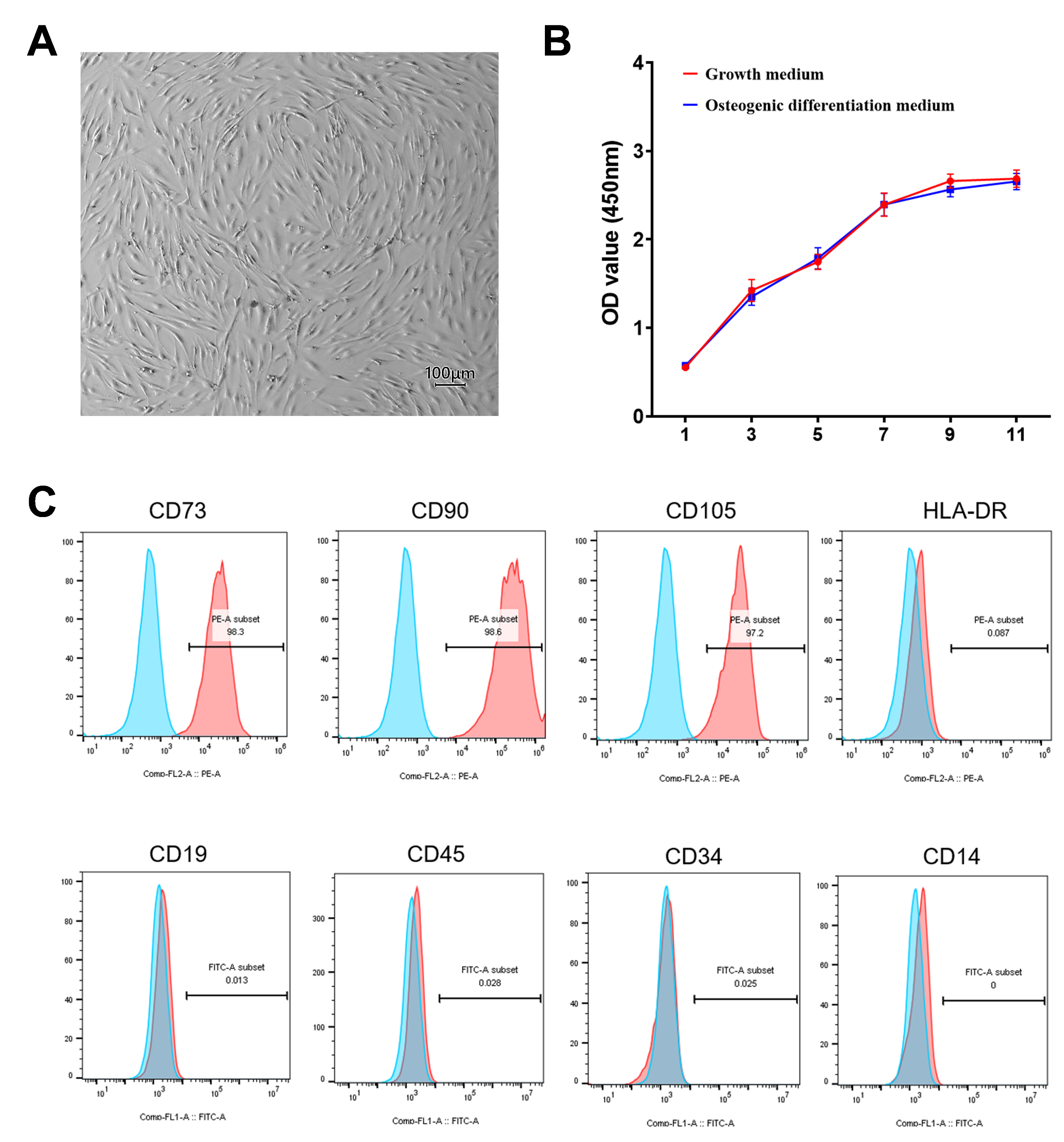


**Figure-S1 Morphology, phenotype, and proliferation rates of AS-MSCs.**

A. Morphological features of AS-MSCs was assessed by microscopy. The scale bar represents 100 μm. B. Proliferation capacities were compared between growth and osteogenic medium. n = 4 samples per group. C. Phenotypes of AS-MSCs were determined by flow cytometry, including CD44, CD73, CD90, CD105, CD14, CD34, CD45 and HLA-DR expression. PE = phycoerythrin; FITC = fluorescein isothiocyanate.

Figure-S2


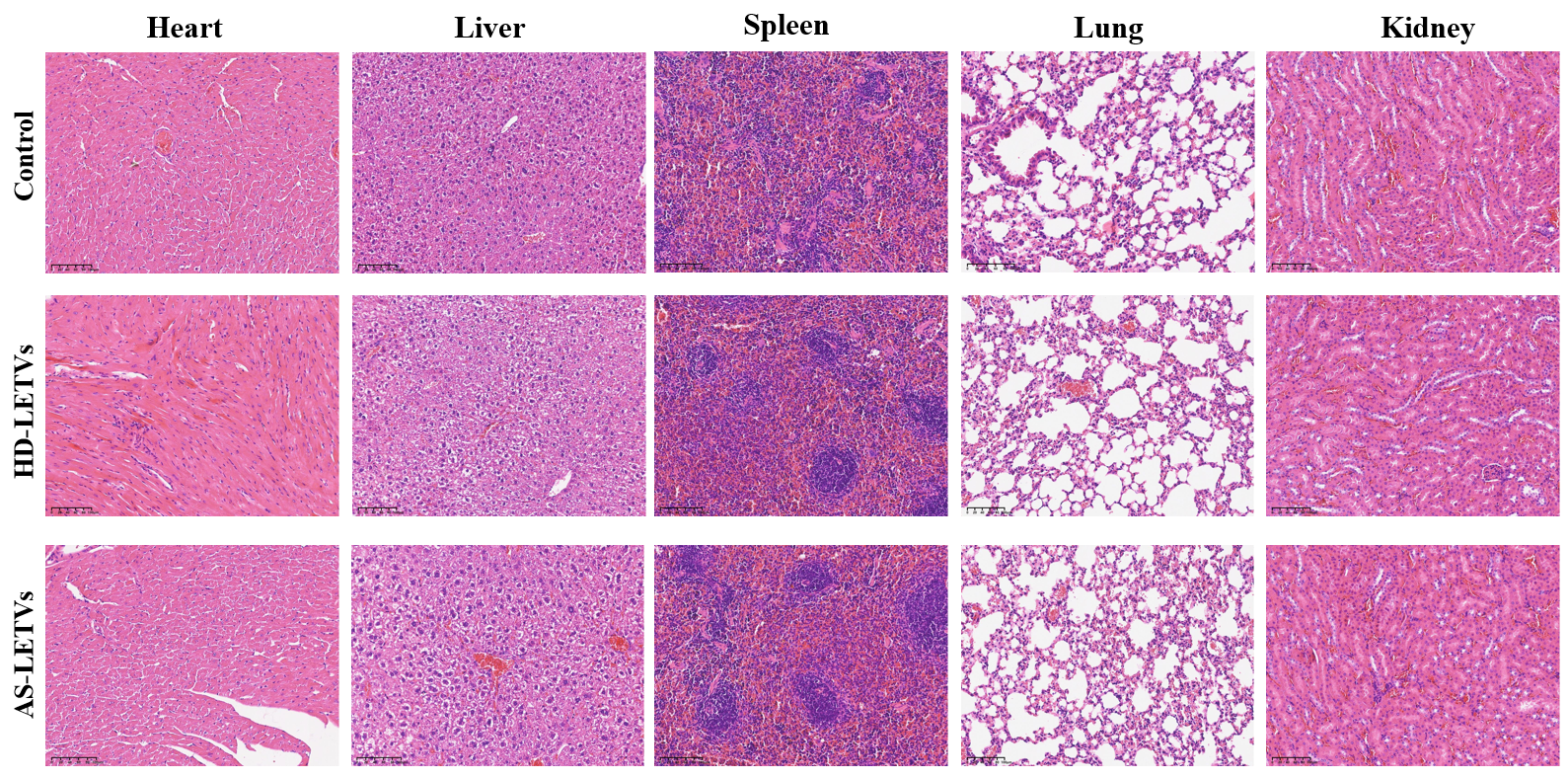


**Figure-S2** Histological evaluation of systemic toxicity in vivo after the indicated treatments Representative images of HE staining of major organs (heart, liver, spleen, lung, kidney) after 4-week serial injections (once weekly) of EVs.

Figure-S3


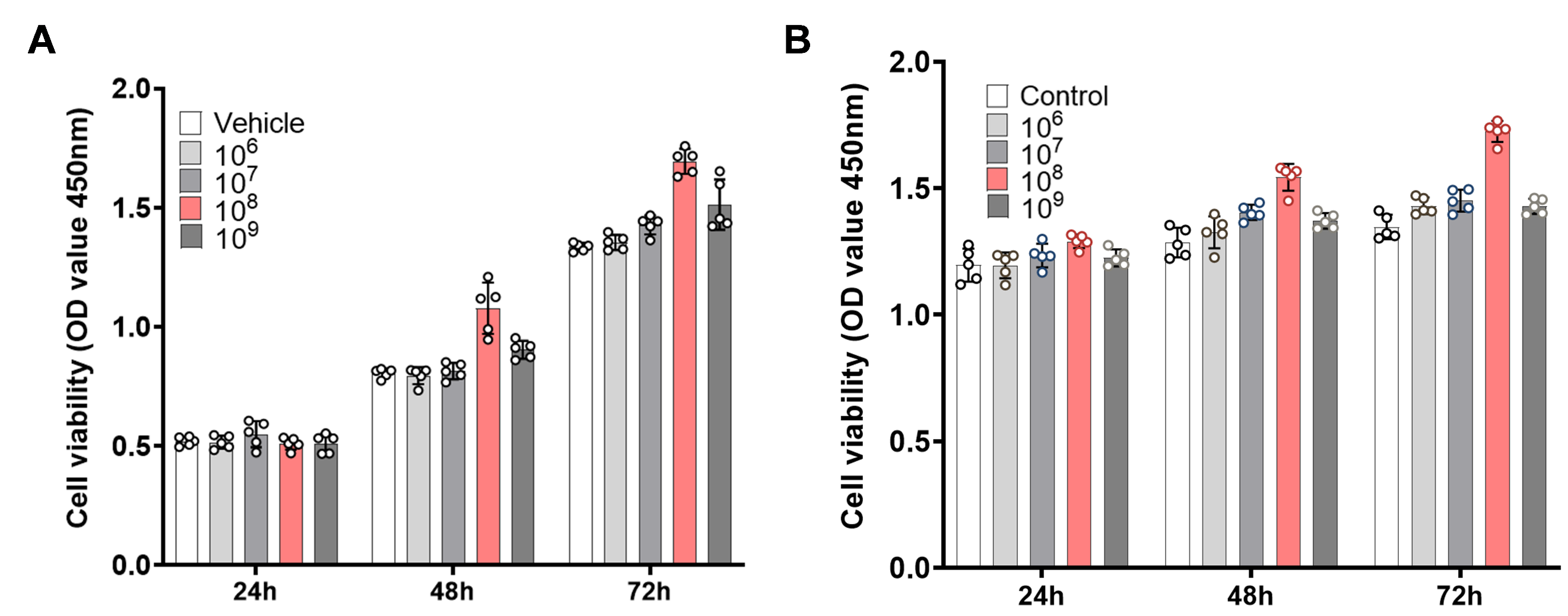


**Figure-S3 Detection of Optimal Concentration for EVs Function.**

To determine the optimal concentration for EVs treatment of MSCs and BBMCs, we conducted CCK-8 assays at different time points and concentrations. A. The concentration of 1×10^8^ particles/mL, EVs exhibited the strongest impact on the viability of both MSCs and BBMCs. This concentration was chosen for subsequent experiments investigating the effects of AS-LTEVs on MSCs and BBMCs. n = 5 samples per group.

Figure-S4


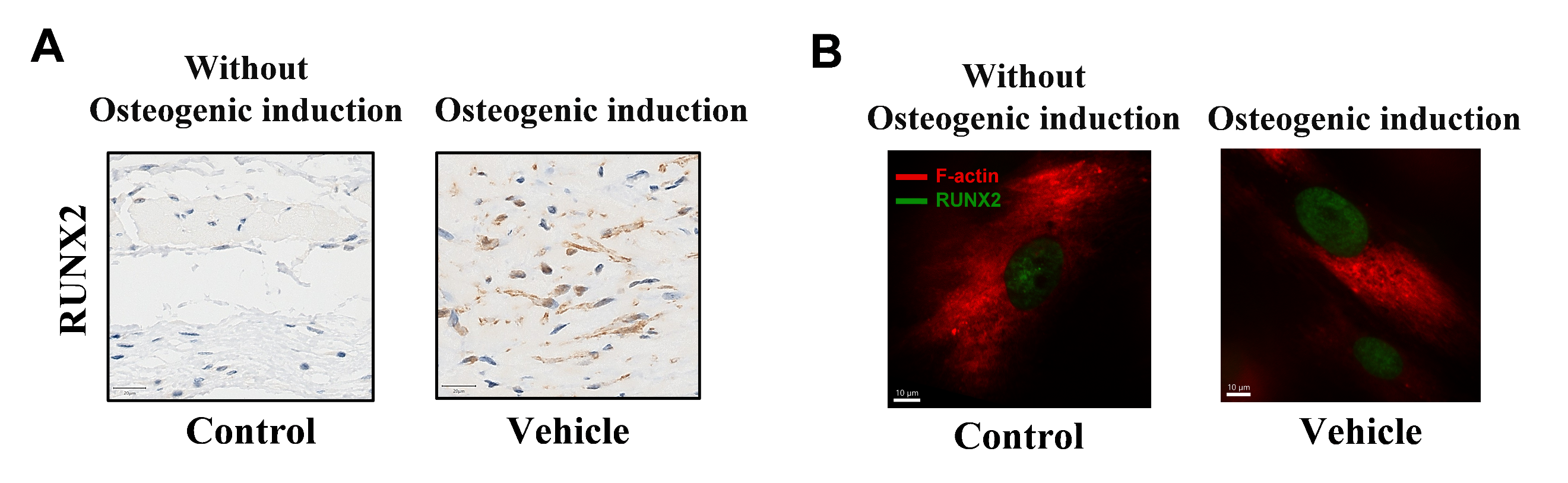


**Figure-S4 Osteogenic induction of AS-MSCs enhanced expression of RUNX2 both *in vitro* and *in vivo*. A.** Transplanting AS-MSCs into nude mice after osteogenic induction, followed by an immunohistochemical assay. Scale bar: 20 μm. B. Immunofluorescence staining was performed to analyze the expression of RUNX2 in AS-MSCs before and after osteogenic induction. Scale bar: 10 μm.

Figure-S5


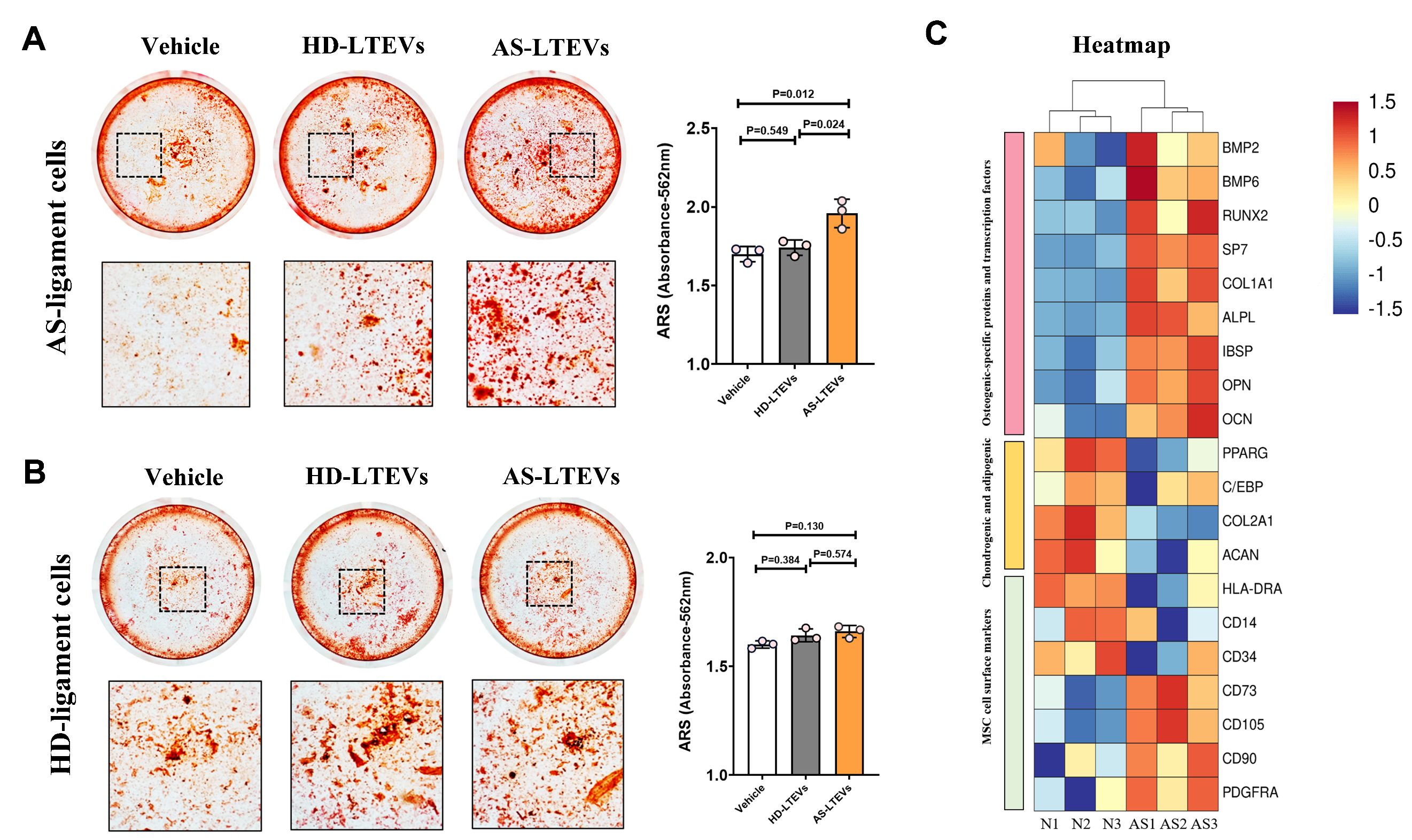


**Figure-S5 AS-LTEVs induce osteogenic differentiation of AS-ligament cells.** Ligament cells obtained from ligament tissue treated with collagenase I and co-cultured with LTEVs. A. AS-LTEVs can promote osteogenesis of AS ligament tissue cells compare with HD-LETVs. n=3 per group. B. LETVs cannot promote osteogenic differentiation of HD ligament cells. n=3 per group. C. The heatmap demonstrates increased expression of osteogenic-specific proteins and transcription factors (BMP, RUNX2, SP7, COL1A1, ALPL, IBSP, OPN, OCN) in AS ligaments. In comparison to HD ligaments, AS ligaments exhibit decreased expression of adipogenic (PPARG, C/EBP) and chondrogenic-related (ACAN, COL2A1) transcription factors, along with increased expression of mesenchymal stem cell surface markers (CD73, CD90, CD105, PDGFRA).

Figure-S6


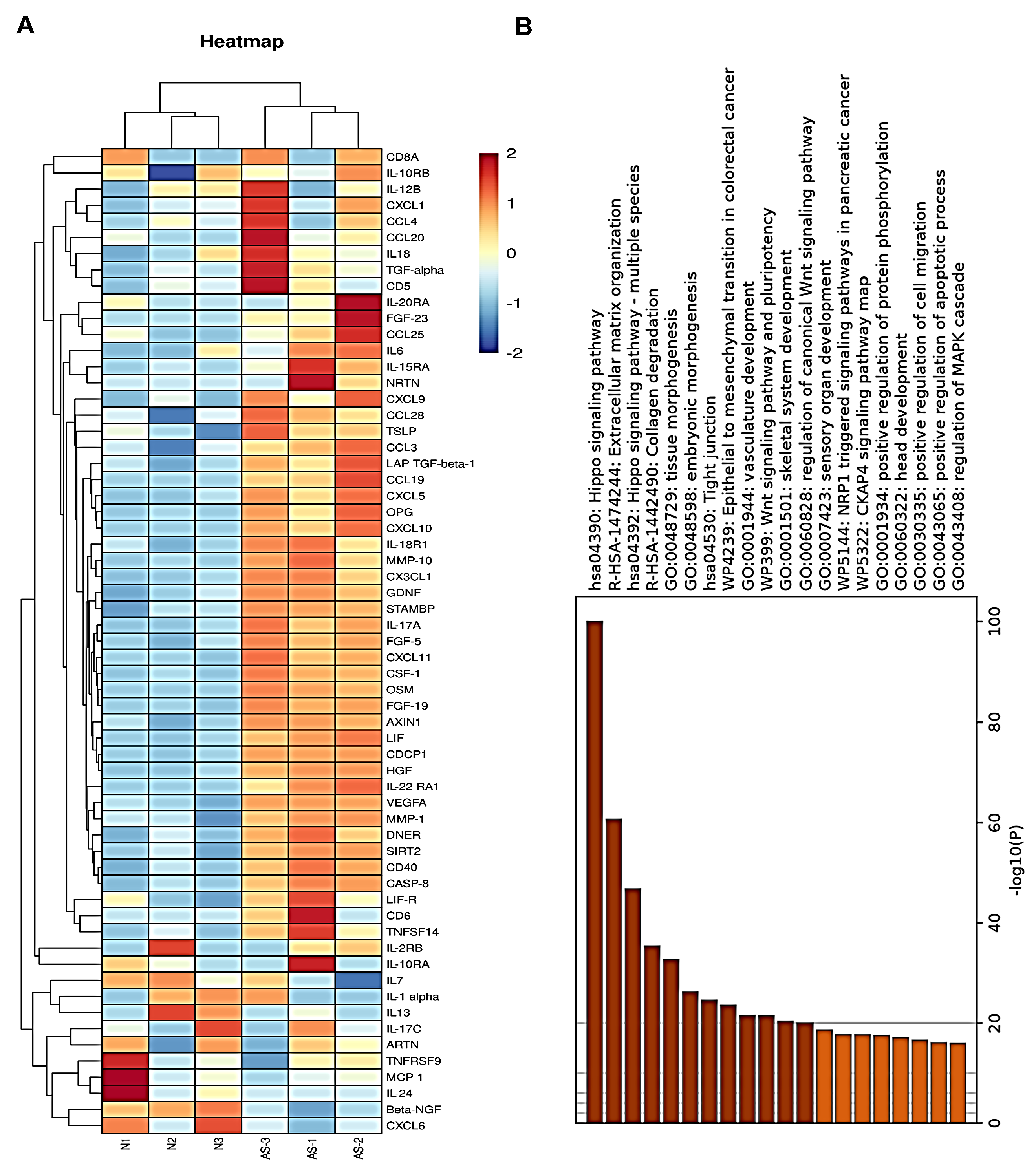


**Figure-S6** Enrichment Analysis show the top 20 statistically enriched terms.

Figure-S7


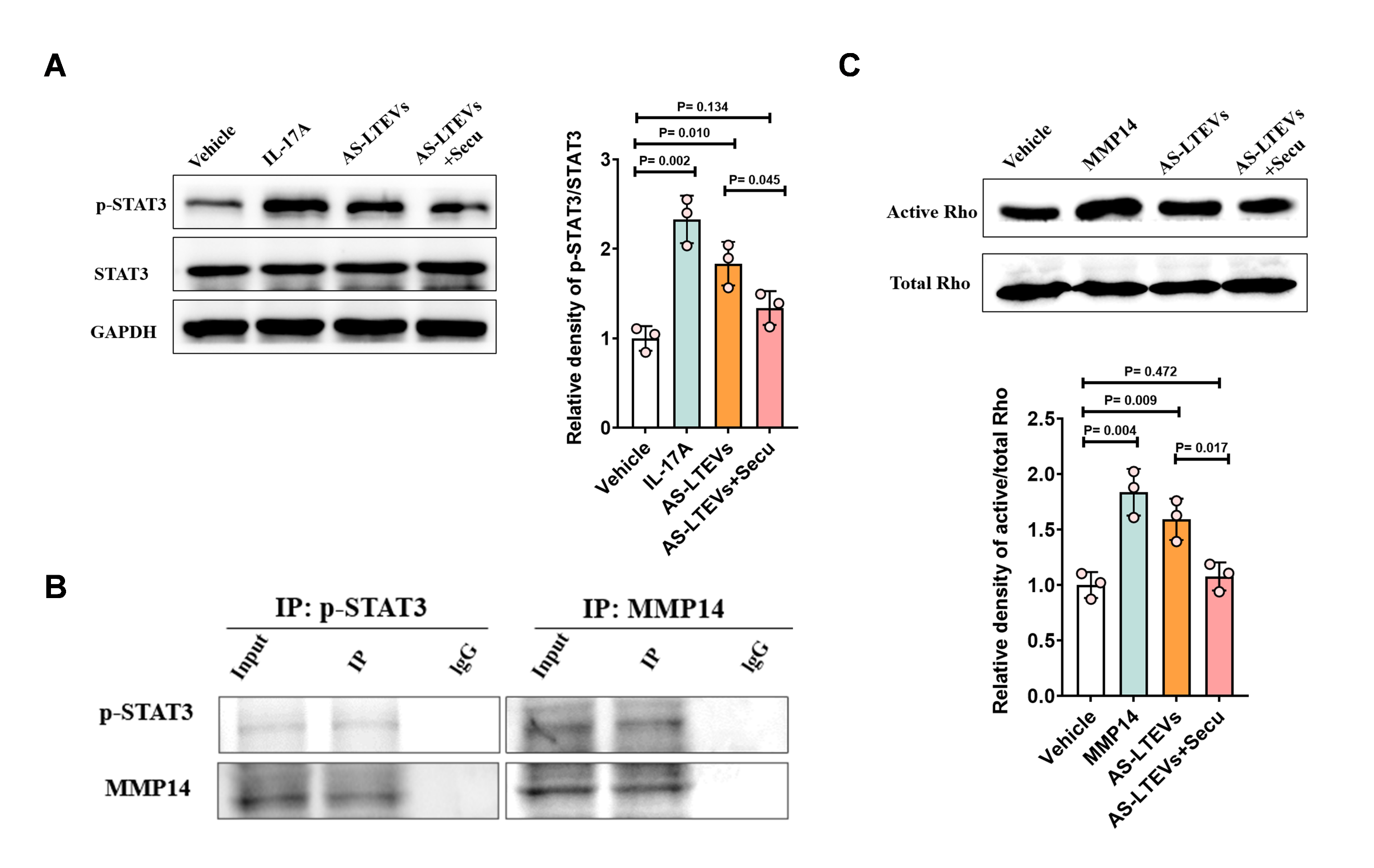


**Figure-S7 AS-LTEV can activate the JAK/STAT3 and further to mediate the Rho/ROCK** **signaling pathway.**

A. AS-LTEVs and IL-17A can activate the JAK/STAT pathway by inducing phosphorylation of STAT3. n = 3 samples per group B. Co-IP experiments results indicate that phosphorylzated-STAT3 can interact with MMP14 during without IL-17A intervention. C. AS-LTEVs and MMP14 can activate the Rho/ROCK signaling pathway, and it can be inhibited by secukinumab. n = 3 samples per group.

Figure-S8


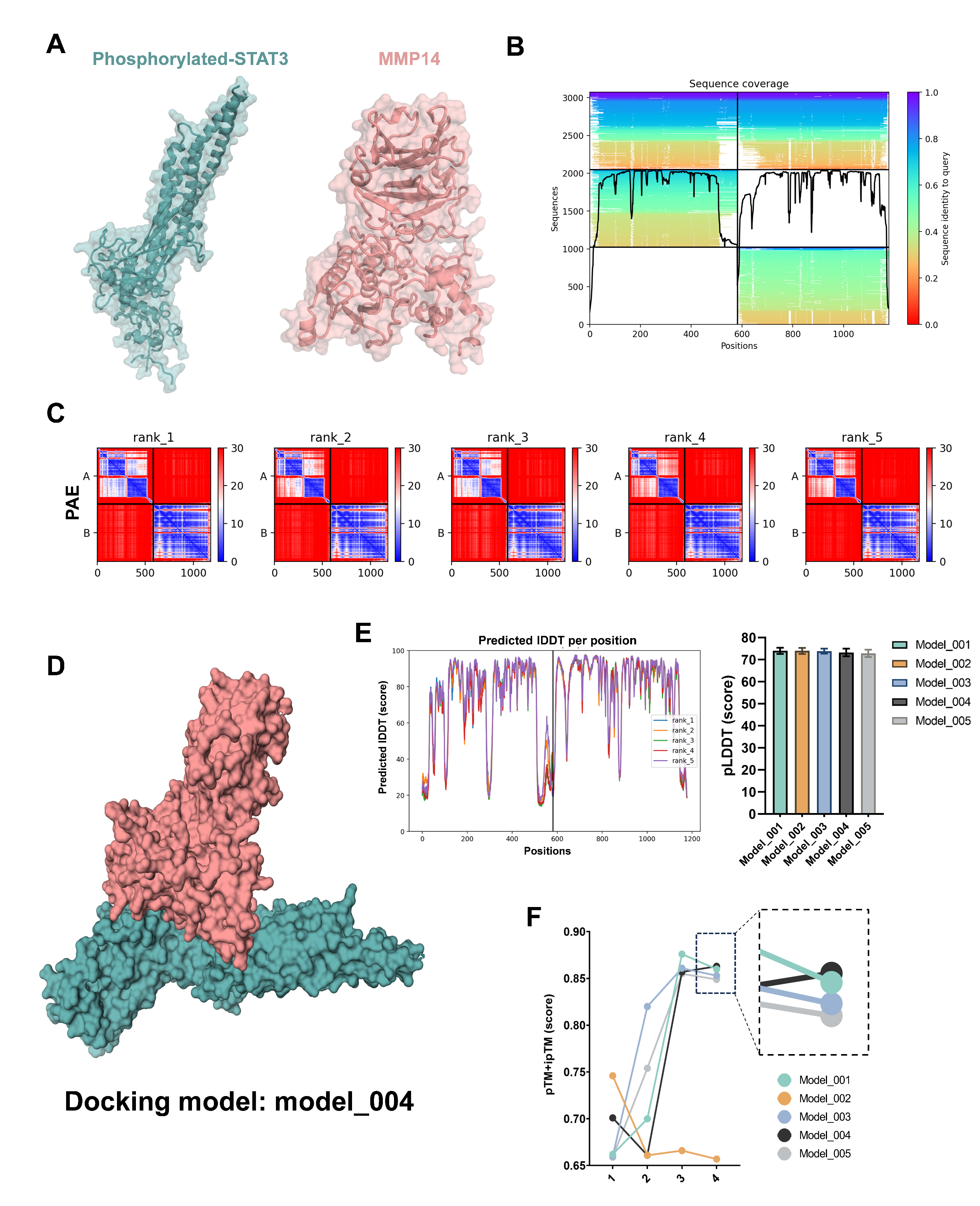


**Figure-S8** **utilizes** **ColabFold tools to predict protein interaction structures and conduct confidence analysis.**

A. Protein structures of Phosphorylated-STAT3 and MMP14 from the protein database are presented. B. Sequence coverage illustrates the abundance of different parts of the input sequence in the protein database. C. Predicted Aligned Error forecasts the alignment error of residue pairs x and y from two proteins. A lower alignment error indicates accurate prediction of their relative positions by AlphaFold. D. The docking model of Phosphorylated-STAT3 and MMP14 is displayed. E. Predicted Local Distance Difference Test (pLDDT) is utilized to assess the accuracy of predicted structures. pLDDT scores provide a measure of confidence in the model predictions, with regions between 70 and 90 being well-modeled. The structural confidence of the five predicted models is high. F. Predicted Total Modeling Score (pTM) reflects the quality of the model prediction for the entire protein interaction sequence, while predicted per-residue confidence (ipTM) provides a measure of confidence in the prediction of each residue. pTM and ipTM are scores for predicting interactions, and a sum >0.75 indicates high confidence in the protein interaction. Among the five predicted models, model_004 exhibits the highest predicted model score for protein interaction confidence.

Figure-S9


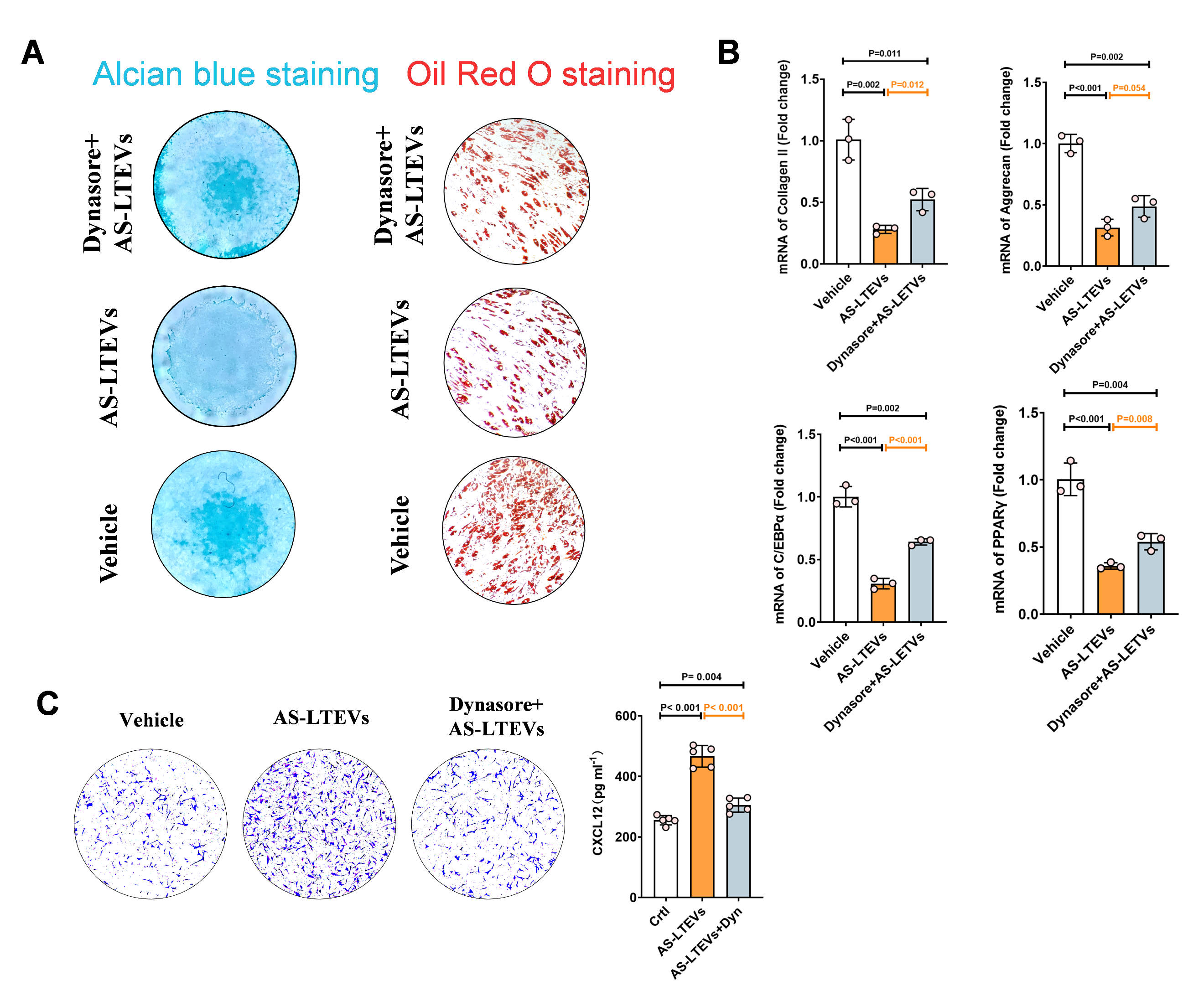


**Figure-S9 AS-LTEVs can promote migration of AS-MSCs while inhibiting adipogenic and chondrogenic differentiation.** A. The Alcian blue staining and Oil Red O staining experiments indicate that AS-LTEVs can inhibit the chondrogenic and adipogenic differentiation of AS-MSCs, inhibiting the endocytosis of AS-LTEVs can improve Alcian blue staining and Oil Red O staining.B. AS-LTEVs can inhibit the expression of adipogenic genes (PPARγ and C/EBPα) and chondrogenic genes (Collagen II and Aggrecan) in AS-MSCs, and inhibiting the endocytosis of AS-LTEVs can promote the expression of adipogenic genes and chondrogenic genes. Scale bar: 100 μm, n=3 per group. C. The transwell results shows that AS-LTEVs can promote the cell migration of AS-MSCs, and the migration promoting effect of AS-LTEVs can be reversed by EV endocytosis inhibitors. Scale bar: 500 μm, n=5 per group.

Figure S10

**
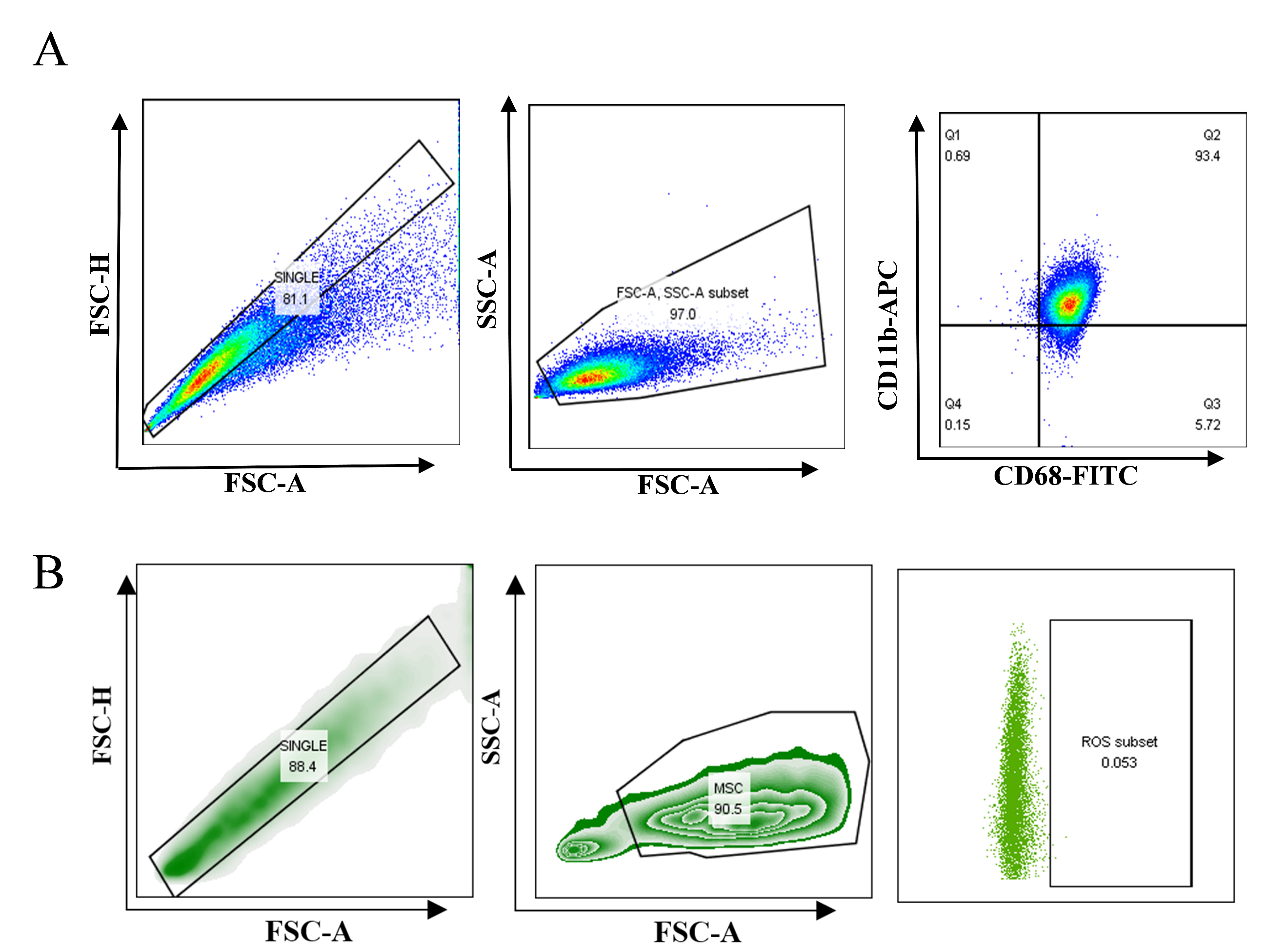
**

Figure S10. A. Experimental scheme of macrophage polarization gating-strategies. B. Experimental scheme of gating-strategies of ROS in BBMCs.

Figure-S11


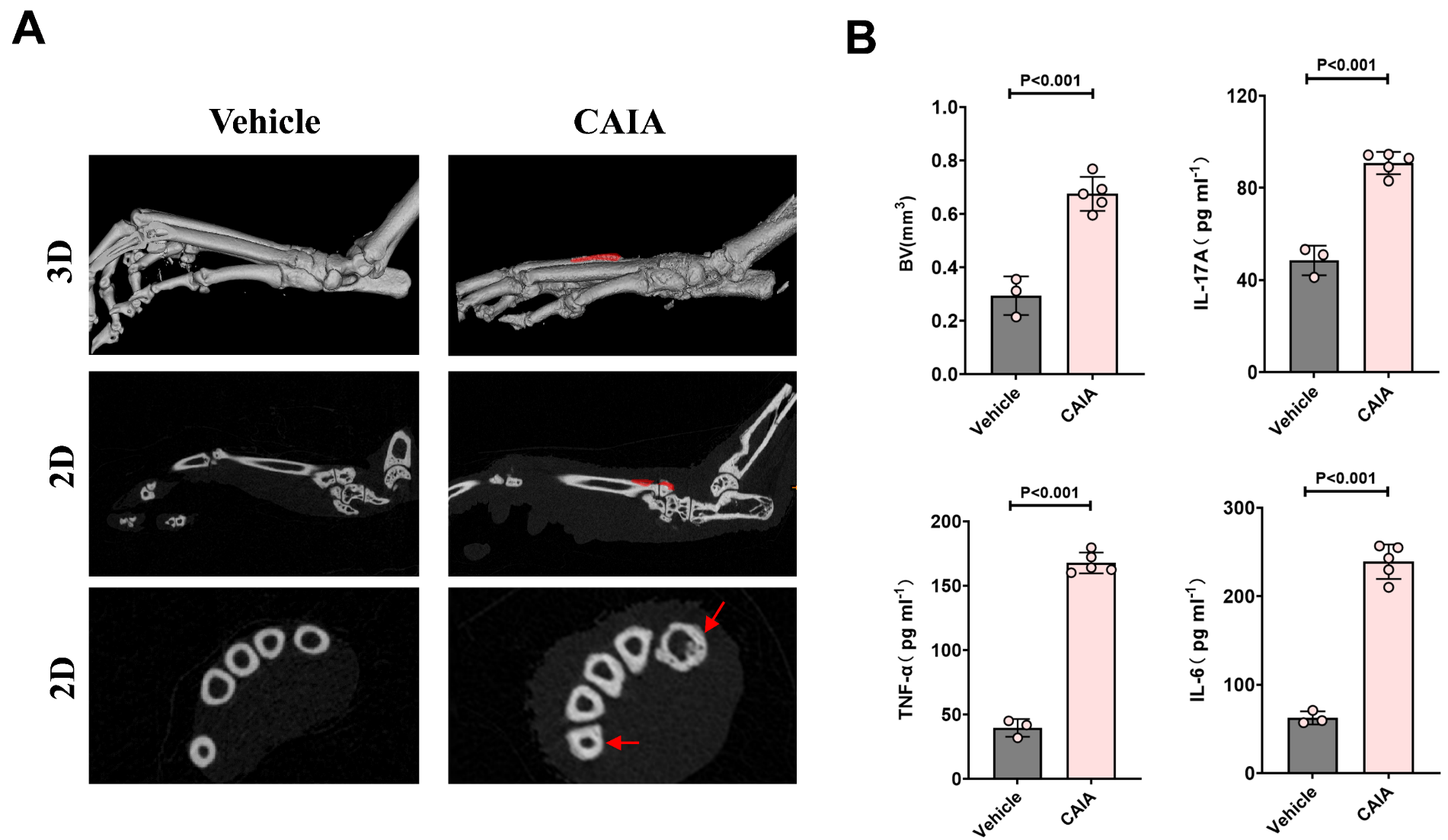


**Figure-S11 The CAIA model is characterized by acute inflammatory responses and pathological ossification.** A. The result of μCT analysis showed that from 2D to 3D cross-sectional images reveals pronounced pathological new bone formation at the paw in the CAIA model. B. ELISA results showed that serum inflammation factor, TNF-α, IL-17A, IL-6, were increased in CAIA model.

Figure-S12


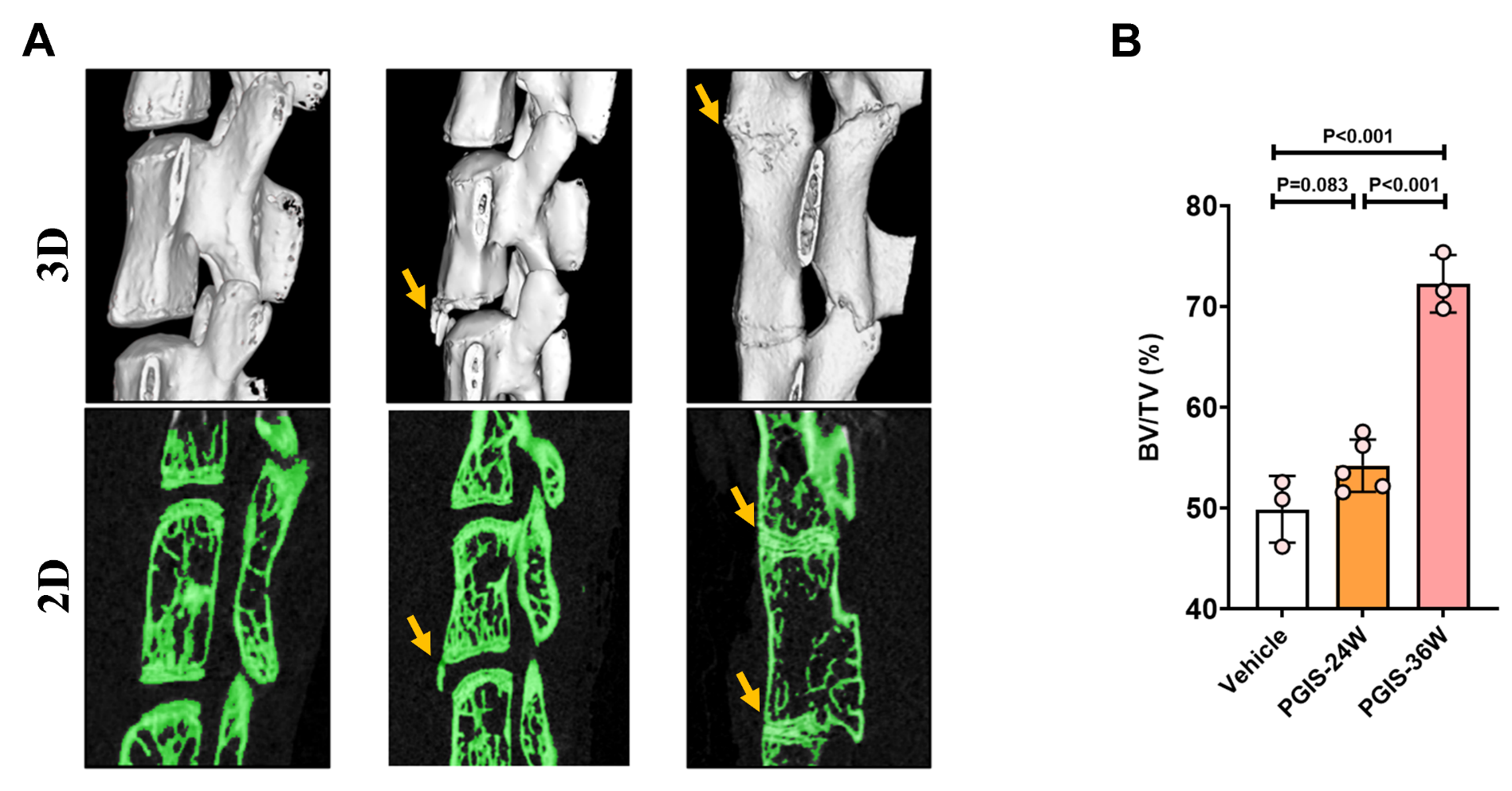


**Figure-S12** The PGIS animal model μCT results show that there is a gradual onset of pathological new bone formation in the spine at 24 weeks, progressing to segmental pathological fusion of the spine by 36 weeks.

| Table S1:  Demographic and clinical data of patients of source of MSCs. | | |
| --- | --- | --- |
|  | Non-AS  N=5 | AS  N=5 |
| Sample | bone  ligamentum flavum  supraspinatus ligament  interspinous ligament | bone  ligamentum flavum  supraspinatus ligament  interspinous ligament |
| Age | 36.2±6.55 | 37.4±7.81 |
| SEX(M/F) | 3/2 | 3/2 |
| BASDAI | 0 | 5.4±1.02 |
| BASFI | 0 | 6.2±0.75 |
| HLA-B27(+/-) | 0/5 | 5/0 |
| Treatment before surgery | NSAIDs | NSAIDs |
| Main diagnosis | Spine fracture | AS |
| Surgical indication | Neurological Compression | Severe Kyphosis |
| \| Table S2: Demographic and clinical data of patients’ tissues were to the extract EVs. \| \| \| \| --- \| --- \| --- \| \|  \| Non-AS  N=10 \| AS  N=20 \| \| Sample \| ligamentum flavum  supraspinatus ligament  interspinous ligament \| ligamentum flavum  supraspinatus ligament  interspinous ligament \| \| Age \| 38.1±5.11 \| 39.4±5.83 \| \| SEX(M/F) \| 5/5 \| 12/8 \| \| BASDAI \| 0 \| 5.6±1.07 \| \| BASFI \| 0 \| 6.1±0.83 \| \| HLA-B27(+/-) \| 0/10 \| 20/0 \| \| Treatment before surgery \| NSAIDs \| NSAIDs \| \| Main diagnosis \| Spine fracture \| AS \| \| Surgical indication \| Neurological Compression \| Severe Kyphosis \|   AS: Ankylosing Spondylitis, BASDAI: Bath Ankylosing Spondylitis Disease Activity Index. BASFI: Bath Ankylosing Spondylitis Functional Index. | | |

Table S3. Inhibitor cocktails used to control proteolysis during homogenization of ligament tissue

| Protease inhibitors  (working concentration) | Target protease type | Stock solution |
| --- | --- | --- |
| PMSF (0.2 mM) | Serine | PMSF: 200 mM in ethanol or isopropanol |
| Benzamidine (1 mM) | Serine | 100 mM in aqueous solution |
| Leupeptin (10 µg/mL) | Serine/cysteine | 1 mg/mL in aqueous solution |
| Pepstatin (10 µg/mL) | Aspartic | 5 mg/mL in methanol |
| Aprotinin (1 µg/mL) | Serine | 0.1 mg/mL in aqueous solution |
| EDTA (1 mM) | Metallo | 100 mM in aqueous solution |

PMSF: phenylmethylsulfonyl fluoride. EDTA: ethylenediamine tetraacetic acid

Table S4. Primer sequences for RT-qPCR (from 5’ to 3’ terminal).

| **Gene** | **Species** | **Forward Primer Sequence** | **Reverse Primer Sequence** |
| --- | --- | --- | --- |
| β-actin | Human | TGACGTGGACATCCGCAAAG | CTGGAAGGTGGACAGCGAGG |
| OCN | Human | CACTCCTCGCCCTATTGGC | CCCTCCTGCTTGGACACAAAG |
| OPN | Human | CTCCATTGACTCGAACGACTC | CAGGTCTGCGAAACTTCTTAGAT |
| RUNX2 | Human | TGGTTACTGTCATGGCGGGTA | TCTCAGATCGTTGAACCTTGCTA |
| OSX | Human | CCTCTGCGGGACTCAACAAC | AGCCCATTAGTGCTTGTAAAGG |
| IL-17A | Human | CTGGAGGATAACACTGTGAGAGT | TGCTGAATGGCGACGGAGTTC |
| ACAN | Human | ACTCTGGGTTTTCGTGACTCT | ACACTCAGCGAGTTGTCATGG |
| COL II | Human | TGGACGATCACGAAACC-3 | G CTGCGGATGCTCTCAATCT |
| PPARγ | Human | AGGCCATTTTCTCAAACGAG | CCATTACGGAGAGATCCACG |
| C/EBPα | Human | ATTGCCTAGGAACACGAAGCACGA | TTTAGCAGAGACGCGCACATTCAC |
| OCN: Osteocalcin, OPN: Osteopontin, OSX: Osterix, ACAN: Aggrecan, COLII: Collagen II | | | |

Table S5: The information of primary anti-body.

| **Antibody** | **Concentration** | **Supplier** | **Catalogue Number** |
| --- | --- | --- | --- |
| ALIX | 1:2000 | Proteintech | 67715-1-Ig |
| CD63 | 1:2000 | Proteintech | 67605-1-Ig |
| TSG101 | 1:1000 | Cell Signaling Technology | #72312 |
| CD9 | 1:2000 | Proteintech | 60232-1-Ig |
| β-actin | 1:1000 | Cell Signaling Technology | #4970 |
| GAPDH | 1:1000 | Cell Signaling Technology | #5174 |
| RUNX2 | 1:1000 (WB)  1:100 (IHC)  1:100 (IF) | Proteintech | 20700-1-AP |
| OCN | 1:1000 | Abcam | ab133612 |
| IL-17A | 1:2000 | Abcam | ab79056 |
| MMP14 | 1:1000 (WB)  1:100 (IHC)  1:200 (IF, FC) | Abcam | ab51074 |
| STAT3 | 1:1000 | Proteintech | 60199-1-Ig |
| Phosphorylated-STAT3 | 1:1000 (WB)  1:500 (IHC) | Abcam | ab267373 |
| YAP1 | 1:1000 | Proteintech | 66900-1-Ig |
| Phosphorylated-YAP (phospho S127) | 1:1000 (WB)  1:100 (IHC)  1:200 (IF) | Abcam | ab76252 |
| LATS1 | 1:1000 | Cell Signaling Technology | #9135 |
| Phosphorylated- LATS1 | 1:1000 | Cell Signaling Technology | #9157 |
| CD73 | 5 µL (0.125 µg)/test | eBioscience | 12-0739-42 |
| CD90 | 5 µL (0.25 µg) /test | eBioscience | 12-0909-42 |
| CD105 | 5 µL (1 µg)/test | eBioscience | 12-1057-42 |
| HLA-DR | 5 µL (0.007 µg)/test | eBioscience | 12-9956-42 |
| CD19 | 5 µL (1 µg)/test | eBioscience | 11-0199-42 |
| CD45 | 5 µL (0.25 µg)/test | eBioscience | 11-0459-42 |
| CD34 | 5 µL (0.5 µg)/test | eBioscience | 11-0349-42 |
| CD14 | 5 µL (1 µg)/test | eBioscience | 11-0149-42 |
| CD68 | 5 µL (0.125 µg)/test | eBioscience | 11-0689-42 |
| CD11b | 5 µL (0.5 µg)/test | eBioscience | 17-0118-42 |
| CD86 | 5 µL (0.25 µg)/test | eBioscience | 46-0869-42 |
| MHCII | 5 µL (0.02 µg)/test | eBioscience | 12-5321-82 |

| Table S6. Top 20 clusters with their representative enriched terms (one per cluster). | | | | | | |
| --- | --- | --- | --- | --- | --- | --- |
| **GO** | **Category** | **Description** | **Count** | **(%)** | **Log10(P)** | **Log10(q)** |
| hsa04390 | KEGG Pathway | Hippo signaling pathway | 62 | 36.47 | -100.00 | -95.66 |
| R-HSA-1474244 | Reactome Gene Sets | Extracellular matrix organization | 51 | 30.00 | -65.65 | -56.61 |
| hsa04392 | KEGG Pathway | Hippo signaling pathway -multiple species | 23 | 13.53 | -46.78 | -43.13 |
| R-HSA-1442490 | Reactome Gene Sets | Collagen degradation | 23 | 13.53 | -35.36 | -32.02 |
| GO:0048729 | GO Biological Processes | tissue morphogenesis | 41 | 24.12 | -32.70 | -29.50 |
| GO:0048598 | GO Biological Processes | embryonic morphogenesis | 36 | 21.18 | -26.23 | -23.18 |
| hsa04530 | KEGG Pathway | Tight junction | 23 | 13.53 | -24.53 | -21.51 |
| WP4239 | Wiki Pathways | Epithelial to mesenchymal transition | 22 | 12.94 | -23.51 | -20.53 |
| GO:0001944 | GO Biological Processes | in colorectal cancer | 31 | 18.24 | -21.48 | -18.67 |
| WP399 | Wiki Pathways | Wnt signaling pathway and pluripotency | 18 | 10.59 | -21.44 | -18.64 |
| GO:0001501 | GO Biological Processes | skeletal system development | 29 | 17.06 | -20.34 | -17.57 |
| GO:0060828 | GO Biological Processes | regulation of canonical wnt signaling pathway | 23 | 13.53 | -20.00 | -17.25 |
| GO:0007423 | GO Biological Processes | sensory organ development | 29 | 17.06 | -17.62 | -15.92 |
| WP5144 | WikiPathways | NRP1 triggered signaling pathways in pancreatic cancer | 13 | 7.65 | -17.62 | -15.00 |
| WP5322 | WikiPathways | CKAP4 signaling pathway map | 16 | 9.41 | -17.50 | -15.00 |
| GO:0001934 | GO Biological Processes | positive regulation of protein phosphorylation | 28 | 16.47 | -17.07 | -14.89 |
| GO:0060322 | GO Biological Processes | Head development | 31 | 18.24 | -17.07 | -14.51 |
| GO:0030335 | GO Biological Processes | positive regulation of cell migration | 27 | 15.88 | -16.63 | -14.01 |
| GO:0043065 | GO Biological Processes | positive regulation of apoptotic process | 26 | 15.29 | -16.06 | -13.57 |
| GO:0043408 | GO Biological Processes | regulation of MAPK cascade | 28 | 16.47 | -15.94 | -13.46 |
| Table S6. Top 20 clusters with their representative enriched terms (one per cluster). "Count" is the number of genes in the user-provided lists with membership in the given ontology term. "%"is the percentage of all of the user-provided genes that are found in the given ontology term (only input genes with at least one ontology term annotation are included in the calculation). "Log10(P)" is the p-value in log base 10. "Log10(q)" is the multi-test adjusted p-value in log base 10. | | | | | | |
